# Longitudinal multi-omics study of palbociclib resistance in HR-positive/HER2-negative metastatic breast cancer

**DOI:** 10.1186/s13073-023-01201-7

**Published:** 2023-07-20

**Authors:** Yeon Hee Park, Seock-Ah Im, Kyunghee Park, Ji Wen, Kyung-Hun Lee, Yoon-La Choi, Won-Chul Lee, Ahrum Min, Vinicius Bonato, Seri Park, Sripad Ram, Dae-Won Lee, Ji-Yeon Kim, Su Kyeong Lee, Won-Woo Lee, Jisook Lee, Miso Kim, Hyun Seon Kim, Scott L. Weinrich, Han Suk Ryu, Tae Yong Kim, Stephen Dann, Yu-Jin Kim, Diane R. Fernandez, Jiwon Koh, Shuoguo Wang, Song Yi Park, Shibing Deng, Eric Powell, Rupesh Kanchi Ravi, Jadwiga Bienkowska, Paul A. Rejto, Woong-Yang Park, Zhengyan Kan

**Affiliations:** 1 Department of Medicine, Samsung Medical Center, Sungkyunkwan University School of Medicine, Seoul, Republic of Korea; 2 Department of Health Science and Technology, School of Medicine & SAIHST, Sungkyunkwan University, Seoul, Republic of Korea; 3 Seoul National University Hospital, Cancer Research Institute, Seoul National University College of Medicine, Seoul National University, Seoul, Republic of Korea; 4 Samsung Genome Institute, Samsung Medical Center, Seoul, Republic of Korea; 5 Oncology Research & Development, Pfizer Inc, San Diego, CA USA; 6 Biostatistics, Pfizer Inc, San Diego, CA USA; 7 Drug Safety R&D, Pfizer Inc, San Diego, CA USA; 8 Research Center for Future Medicine, Samsung Medical Center, Seoul, Republic of Korea; 9 Pfizer Oncology, Seoul, Republic of Korea

**Keywords:** Advanced breast cancer, Drug resistance, Genomic profile, Gene expression profiling, Palbociclib, Homologous recombination repair deficiengy (HRD)

## Abstract

**Background:**

Cyclin-dependent kinase 4/6 inhibitor (CDK4/6) therapy plus endocrine therapy (ET) is an effective treatment for patients with hormone receptor-positive/human epidermal receptor 2-negative metastatic breast cancer (HR+/HER2− MBC); however, resistance is common and poorly understood. A comprehensive genomic and transcriptomic analysis of pretreatment and post-treatment tumors from patients receiving palbociclib plus ET was performed to delineate molecular mechanisms of drug resistance.

**Methods:**

Tissue was collected from 89 patients with HR+/HER2− MBC, including those with recurrent and/or metastatic disease, receiving palbociclib plus an aromatase inhibitor or fulvestrant at Samsung Medical Center and Seoul National University Hospital from 2017 to 2020. Tumor biopsy and blood samples obtained at pretreatment, on-treatment (6 weeks and/or 12 weeks), and post-progression underwent RNA sequencing and whole-exome sequencing. Cox regression analysis was performed to identify the clinical and genomic variables associated with progression-free survival.

**Results:**

Novel markers associated with poor prognosis, including genomic scar features caused by homologous repair deficiency (HRD), estrogen response signatures, and four prognostic clusters with distinct molecular features were identified. Tumors with *TP53* mutations co-occurring with a unique HRD-high cluster responded poorly to palbociclib plus ET. Comparisons of paired pre- and post-treatment samples revealed that tumors became enriched in APOBEC mutation signatures, and many switched to aggressive molecular subtypes with estrogen-independent characteristics. We identified frequent genomic alterations upon disease progression in *RB1*, *ESR1, PTEN*, and *KMT2C*.

**Conclusions:**

We identified novel molecular features associated with poor prognosis and molecular mechanisms that could be targeted to overcome resistance to CKD4/6 plus ET.

**Trial registration:**

ClinicalTrials.gov, NCT03401359. The trial was posted on 18 January 2018 and registered prospectively.

**Supplementary Information:**

The online version contains supplementary material available at 10.1186/s13073-023-01201-7.

## Background

Cyclin-dependent kinase 4/6 inhibitors (CDK4/6i) combined with endocrine therapy (CDK4/6i plus ET) are a standard treatment for patients with hormone receptor-positive/human epidermal growth factor receptor 2-negative metastatic breast cancer (HR+/HER2− MBC) [1]. However, approximately 25% of patients do not respond to CDK4/6i plus ET and those who do respond eventually progress [2]. Thus, there is a pressing need to identify molecular markers that will help identify patients who will benefit most from treatment with CDK4/6i plus ET, and find therapeutic targets to overcome intrinsic and acquired resistance may improve the treatment of HR+/HER2− MBC.

CDK4/6i block the G1/S phase cell cycle transition by selectively targeting CDK4/6, which governs cell cycle progression via retinoblastoma protein phosphorylation [2]. Preclinical and clinical studies have implicated many cell cycle and oncogenic proteins and genes in CDK4/6i resistance. The loss of retinoblastoma 1 (*RB1*) confers resistance to CDK4/6i [3]. Analysis of tumor samples and circulating tumor DNA (ctDNA) of patients receiving CDK4/6i plus ET have shown loss of *RB1* at baseline or with treatment is associated with shorter progression-free survival (PFS), although such alterations appear uncommon, occurring in < 10% of resistant samples [2, 4–6]. A targeted genomic analysis of 348 pretreatment tumors treated with CDK4/6i plus ET found loss-of-function mutations in *FAT atypical cadherin 1* to be associated with much shortened PFS, although these mutations were only present in 1.7% of the tumor samples [4]. Analysis of ctDNA from 34 patients after progression on palbociclib plus ET identified fibroblast growth factor receptors 1/2 (*FGFR1/2*) activating mutations or amplification in 41% of samples [7]. Consistent with this observation, patients from the MONALEESA-2 trial treated with ribociclib plus ET who had *FGFR1* amplification had shorter PFS compared with patients with wild-type *FGFR1*, and patients with an *FGFR1* gain had shorter PFS in the palbociclib plus fulvestrant and placebo plus fulvestrant groups in the PALOMA-3 study [7, 8]. In a recent whole-exome sequencing (WES) study, alterations in numerous genes including *AKT1* and *ERBB2*, *RAS* pathway activating alterations, *AURKA* and *CCNE2* amplification, and *FGFR2* alterations were identified in tumor biopsies from patients with intrinsic or acquired CDK4/6i resistance [6]. In vitro studies confirmed these genetic alterations could confer resistance to CDK4/6i [6, 9, 10]; however, their contribution to resistance in vivo was not reported. Low baseline *CCNE1* expression was significantly associated with improved PFS in patients treated with palbociclib [11].

The above studies demonstrate the genetically heterogeneous and complex nature of CDK4/6i resistance and highlight the difficulty in identifying predictive biomarkers. Despite the identification of potential genes conferring resistance to CDK4/6i [12], we still lack predictive markers as well as biological insights into why a subset of HR+/HER2− MBC patients respond poorly to the therapy.

Here, we perform a comprehensive genomic and transcriptomic profiling of prospectively collected paired pretreatment and post-progression tumor biopsies from HR+/HER2− MBC patients treated with palbociclib plus ET.

## Methods

### Patient enrollment and sample collection

The study conformed to the principles of the Helsinki Declaration. This study was reviewed and approved by the Institutional Review Board (IRB) of Samsung Medical Center (SMC) and Seoul National University Hospital (SNUH), Seoul, Korea (IRB No. 2017-07-049 for SMC and H-1711-075-900 for SNUH; NCT03401359) with written informed consent for the research use of clinical and genomic data. Patients were diagnosed with histologically confirmed invasive HR+/HER2− BC by IHC. Patients who had recurrent and/or metastatic disease were included in this study and were treated with palbociclib plus an aromatase inhibitor or fulvestrant (with a gonadotropin-releasing hormone agonist for premenopausal patients) at SMC and SNUH from 2017 to 2020. The primary outcome measures were biomarkers of palbociclib resistance in metastatic breast cancer from whole-exome sequencing, RNASeq, circulating tumor DNA, and flow cytometry. The inclusion criterion was hormone receptor-positive, metastatic breast cancer treated by palbociclib with endocrine therapy, and the exclusion criteria were refusal to informed consent and withdrawal to this study. The first and the last patients were enrolled on October 26, 2017, and on May 20, 2020, respectively. Longitudinally paired tumor biopsies and serum from patients with HR+/HER2− MBC treated with palbociclib in combination with ET were prospectively collected to conduct biomarker and molecular profiling analyses. Tumor biopsy and blood samples were obtained at pretreatment, on-treatment (6 weeks and/or 12 weeks), and post-progression. Tumor biopsies were profiled using WES and WTS (RNA-Seq). Matched blood samples were subjected to WES to facilitate somatic mutation detection. Fresh tissue or formalin-fixed paraffin-embedded (FFPE) specimens were collected from 71 recruited patients. Biopsied samples were processed immediately or within 15 min post-acquisition.

WES was performed on baseline (BL) samples from 56 patients but only progressive disease (PD) samples from 23 patients. One reason is that disease progression had not occurred for 28.2% (20/71) of the patients enrolled in our prospective study who were still undergoing palbociclib/ET treatment at the time of this analysis (Additional file 1: Table S1). Patients with WES data for PD samples had a shorter median PFS of 13.3 months (95% CI = 8.3–19) compared with the median PFS of 19 months (95% CI = 12.4–24.4) for patients with WES data for BL samples.

### Pathology review of tumor tissue

Hematoxylin and eosin (H&E)-stained sections from each sample were subjected to independent pathology review to confirm that the tumor specimen was histologically consistent and the presence of enough tumor cells. The percent tumor nuclei, percent necrosis, and other pathology annotations were also assessed. The tumor purity was evaluated by counting invasive tumor nuclei among all cellular nuclei in a given slide.

### WES and RNA-Seq

Pathologists determined tumor purity by reviewing tumor specimens, and samples with low tumor purity (cutoff: 20%) were excluded from sequencing. Genomic DNA was extracted from fresh frozen tissues using the QIAamp DNA Mini Kit (Qiagen) and from FFPE tissues using the ReliaPrep FFPE gDNA Miniprep System (Promega). Genomic DNA from the peripheral blood was extracted using the QIAamp DNA Blood Maxi Kit (Qiagen). Total RNA from fresh-frozen tumor tissues was extracted with an RNeasy Mini Kit (Qiagen) and from FFPE tissues using ReliaPrep™ FFPE Total RNA Miniprep System (Promega) according to the manufacturer’s instructions. The quality and quantity of extracted nucleic acids were evaluated using NanoDrop™ 8000 UV–Vis spectrometer (NanoDrop Technologies Inc.), Qubit® 3.0 Fluorometer (Life Technologies, Inc.), and 4200 TapeStation (Agilent Technologies, Inc.).

High-quality gDNA in matched tumor and blood samples was sheared with an S220 ultra-sonicator (Covaris, Inc.) and used to construct a library with the SureSelect XT Human All Exon v5 and SureSelect XT reagent kit, HSQ (Agilent Technologies, Inc.), according to the manufacturer’s protocol. Libraries were pooled, denatured, and sequenced in 100-bp paired-end mode using the HiSeq Rapid SBS Kit v2 (200 cycles) and HiSeq Rapid PE Cluster Kit v2 in Illumina HiSeq 2500 platforms (Illumina, Inc.). The mean target coverage was 145× for tumor and 100× for blood. Reads were aligned to the human reference genome (hg19) using the Burrows-Wheeler Alignment tool (BWA) v.0.7.17 [13].

Sequence Alignment and Mapping (SAM) files were converted to Binary Alignment and Mapping (BAM) files using SAMtools v1.6 (http://www.htslib.org/). Duplicate reads were removed using Picard v2.9.4 (http://broadinstitute.github.io/picard/), base quality was recalibrated, and local realignment was optimized using the Genome Analysis Toolkit (GATK) v4.0.2.1 [14]. Single nucleotide variants (SNVs) and indels were identified using MuTect2 v4.0.2.1 in GATK. Germline variants were identified using GATK HaplotypeCaller v3.8.0. Variants were annotated using Ensembl Variant Effect Predictor (VEP) version 87 [15]. Copy number alteration was estimated by ExomeCNV R package v1.4 [16]. Tumor purity was inferred from FACETS R package v0.6.0 [17].

To remove low confidence or artifact mutation calls, the mutations for the selected genes were also manually reviewed by Bambino Viewer [18] and Integrative Genomics Viewer (IGV) [19]. A mutation call was considered to be low confidence if it has no high-quality sequencing reads in support of the call or has < 3 supporting mutant reads in all longitudinal samples from the same patient. In addition, mutation calls clustered with other variants were evaluated as a potential paralog alignment artifact and removed if manually confirmed by BLAT on UCSC Genome Browser [20].

Sequencing libraries were prepared with TruSeq RNA Access Library Prep Kit (Illumina, Inc.) from FFPE tissues following the manufacturer’s protocols. Paired-end sequencing of the RNA libraries was performed on a HiSeq 2500 Sequencing Platform (Illumina, Inc.). After trimming poor-quality bases from the FASTQ files, reads were aligned to the human reference genome (hg19) with STAR v2.5.2b [21], and estimated gene expression was calculated in terms of transcripts per million (TPM) using RSEM v1.3 [22]. A pseudocount of one was added to TPM before the log2 transformation. Batch effects between fresh-frozen and FFPE tissues were then corrected on log2 TPM values using the ComBat function from sva R package v3.30.1.

### Immunohistochemistry and digital imaging analysis

Immunohistochemistry (IHC) assays were run using validated protocols. Briefly, 5-µm sections were cut from tumor biopsy specimens and immunolabeled for the following antigens: Cyclin E1 (clone HE12) and pRb (clone #9308; Cell Signaling Technology, Danvers, MA), Cyclin E2 (clone E142; Abcam, Waltham, MA), and Ki67 (clone MIB-1; DAKO, Denmark). Slides were scanned using a Leica Aperio AT2 slide scanner (Leica Biosystems, Vista CA) at × 20 magnification setting. Whole-slide image analysis was performed using the Visiopharm software (Visiopharm, Denmark). Custom algorithms were written to delineate tumor nests from nontumor-viable regions. A pathologist verified the results, and manual corrections were made wherever necessary. Regions containing necrotic tissue, fat, and ductal carcinoma in situ were manually excluded. For each biomarker, a separate workflow was developed to detect nuclei and classify them as either positive or negative for that biomarker. The positive nuclei were then scored as high (3+), medium (2+) or low (1+). The *H*-score was then calculated using the following equation (# denotes number):

$$H-\mathrm{score}= 100\times \frac{3\times (\#\mathrm{ of }3+\mathrm{ nuclei}) + 2\times (\#\mathrm{ of }2+\mathrm{ nuclei}) + (\#\mathrm{ of }1+\mathrm{ nuclei})}{\mathrm{Total number of nuclei}}$$

For each biopsy specimen, *H*-scores were calculated for the tumor nests and for the entire viable tissue (tumor nests + nontumor viable regions), respectively.

### Genomic and molecular features

PAM50 classification was performed using Genefu v2.14.0 [23] and the gene expression data. The proliferative index for each tumor sample was calculated as the geometric mean of gene expression (TPM) using an 11-gene signature: *BIRC5*, *CCNB1*, *CDC20*, *NUF2*, *CEP55*, *NDC80*, *MKI67*, *PTTG1*, *RRM2*, *TYMS*, and *UBE2C* [24]. Tumor mutational burden (TMB) was calculated as the number of protein-altering mutations in each sample, including essential splice site, frameshift, in-frame indel, missense, nonsense, and stop-loss mutations. BRCA1/2 pathogenic mutation was determined considering both germline and somatic mutations that truncate protein reading frame or annotated as pathogenic in ClinVar. Genomic scar features were quantified including large-scale transitions (LST), telomeric allelic instability (TAI), and loss of heterozygosity (HRD-LOH) using allele-specific copy number, tumor purity, and ploidy inferred by FACETS as described previously [25–27]. The HRD index was derived as the unweighted sum of LST, TAI, and HRD-LOH. The relative contribution of each mutational signature in samples was inferred using deconstructSigs v1.8.0 [28], which identifies the linear combination of reference signatures to best explain the mutation profiles observed in the 96-trinucleotide contexts. The gene set variation analysis (GSVA) R package v1.30.0 [29] was used to calculate signature scores for 935 gene sets from molecular signatures database (MsigDB) v5.2 [2] collections H (hallmark), C2 (KEGG, REACTOME), and Bindea and colleagues’ 24 immune signatures [30]. Thirty total signatures with *p* < 0.01 were selected for clustering analysis. A predefined set of 30 mutational signatures (v2) from the Wellcome Trust Sanger Institute was used as reference signatures [31]. Among these, we then identified six signatures present in more than 2 samples with a score > 0.2.

### Integrative clustering analysis

To identify distinct molecular states of the disease, we used iClusterPlus v1.28.0 [32] to cluster and classify BL and PD samples as a joint multivariable regression of tumor-intrinsic molecular markers to identify a set of latent variables representing the underlying disease states. Significant prognostic markers were selected including *BRCA1/2* pathogenic mutation status, PAM50 molecular subtypes, *TP53* mutation status, HRD index, proliferative index, TMB, HRD, and apolipoprotein B editing complex (APOBEC) mutation signatures, and 2 representative expression signatures (HALLMARK E2F TARGETS and HALLMARK ESTROGEN RESPONSE EARLY). The optimal number of clusters was determined based on the Bayesian information criterion.

### Identification of PD-specific genomic alterations

To identify PD-specific genomic alterations, a cohort of 20 patients was assembled with WES data available from paired BL and PD samples, plus 1 patient (BRO7F−008) with WES coverage for paired 6-week and PD samples. A genomic alteration was deemed PD-specific if it was detected in the PD but not in BL or 6-week samples. Only copy number variation (CNV) events were included that were likely to play a functional role by requiring copy number (CN) > 6 and gene expression in the upper quartile of the cohort or with CN < 1.2 and gene expression in the lower quartile. Gene fusions were predicted using STAR-Fusion v1.9.0 [33] and then manually reviewed to identify high-confidence fusion events. Genomic events were aggregated at the gene level to calculate the frequency of PD-specific genomic alterations.

### Data sharing statement

Upon request, and subject to review, Pfizer will provide the data that support the findings of this study. Subject to certain criteria, conditions, and exceptions, Pfizer may also provide access to the related individual de-identified participant data (see https://www.pfizer.com/science/clinical-trials/trial-data-and-results for more information).

### Statistical methods

#### PFS association analysis of clinico-genomic variables and gene signatures

Univariate Cox regression analysis was performed to identify clinical and genomic data variables significantly associated (*p* < 0.05) with PFS at baseline. The statistical significance of the association was assessed using the log-rank test and Kaplan-Meier method using the survival R package v3.2.7 and the survminer R package v0.4.8. Continuous variables were median split into categorical variables. FDR (*q*-value) was calculated using the Benjamini and Hochberg method [34]. All statistical analyses were performed using R version 3.6.1, and *p* < 0.05 was considered to be statistically significant.

Thirteen clinico-genomic variables univariately associated with PFS were used to build a multivariate Lasso regression model for PFS (glmnet R package v4.1-1) [35]. Standardized variable importance metrics (caret R package v6.0-86) [36] were averaged across the re-samplings and then exponentiated to generate independent covariate risks on disease progression (HRs). In the survival elastic net multivariate models, the variable importance value is the absolute standardized *β* estimates.

## Results

### Patient clinical characteristics and tumor attributes

**Fig. 1:**
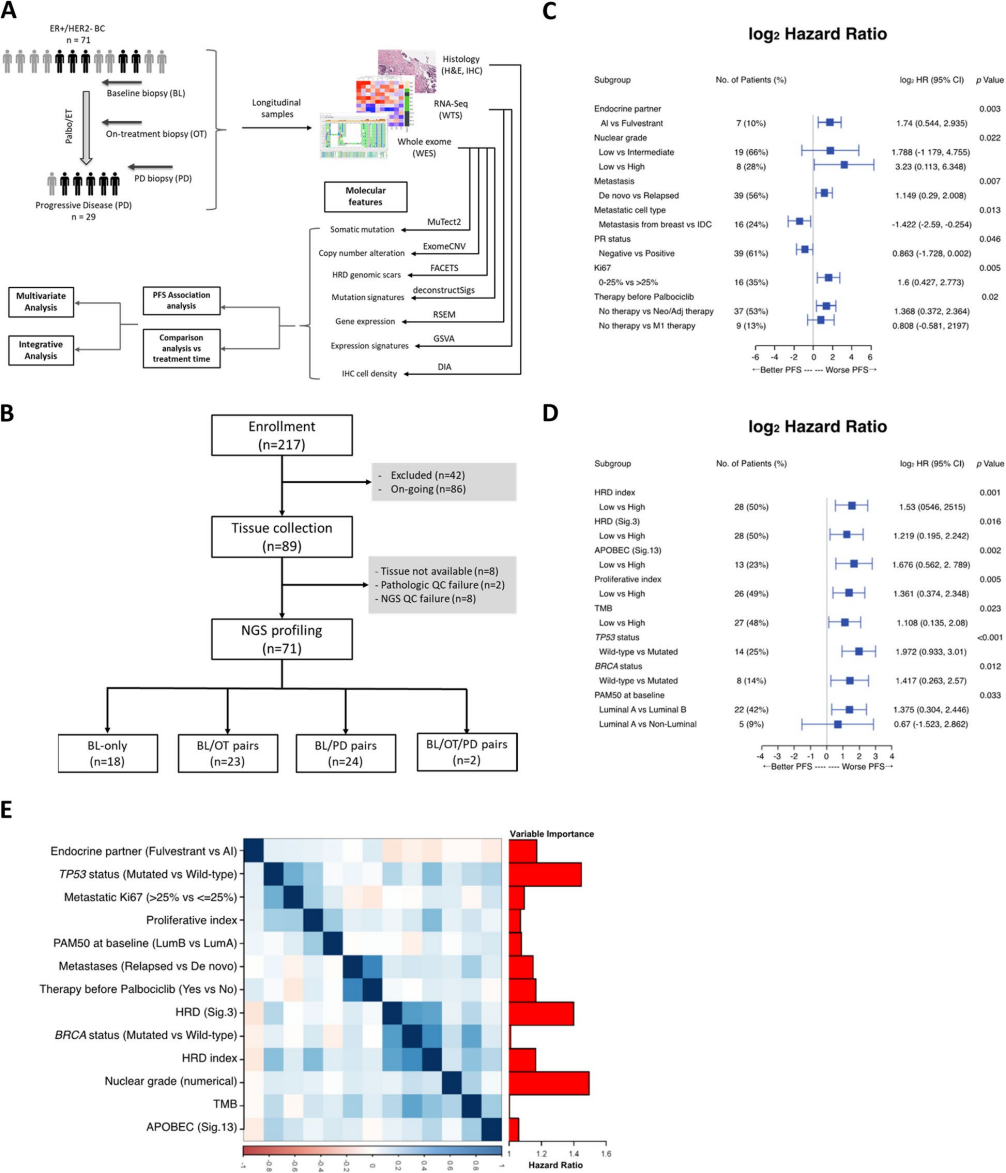
Identification of prognostic markers. **A** Overview of the study design and data analysis. H&E, hematoxylin and eosin; HRD, homologous recombination deficiency. **B** Consort diagram showing patient enrollment and biopsy collection. QC, quality control. NGS profiling statuses for four patients are not shown: OT-only (*n* = 1), OT/PD (*n* = 1), and PD-only (*n* = 2). Forest plots of clinical variables (**C**) and molecular features (**D**) significantly associated with PFS. HR based on PFS calculated using univariate Cox regression analysis and variables with log-rank *P*-value < 0.05 considered significantly different in PFS. Continuous variables divided into high and low groups based on the median. **C** AI: letrozole/letrozole + GnRH/exemestane + GnRH. PR, progesterone receptor; M1, palliative treatment; ILC, invasive lobular carcinoma; IDC, invasive ductal carcinoma. **D** Signature: COSMIC Mutational Signature (version 2). TMB, tumor mutation burden; PAM50, intrinsic breast cancer subtype; non-luminal, HER2-enriched, basal, or normal-like subtype. **E** Correlogram (center) among clinical and molecular features significantly associated with PFS shows clusters of highly correlated covariates. Averages of variable importance for increased risk of progression (vertical right) based on 500 random resamples of survival elastic net models (75% training sets) show *TP53* status, HRD (S3), and nuclear grade as the most important predictors of progression. In survival elastic net multivariate models, variable importance value is absolute standardized beta estimates

The study design is shown in Fig. 1a. In total, 217 patients with HR+/HER2− MBC receiving palbociclib plus ET were enrolled, and next-generation sequencing (NGS) profiling was successfully conducted on biopsies from 71 patients (Fig. 1b). There were 56 baseline (BL) biopsies profiled by WES and 64 by whole transcriptome sequencing (WTS); 23 progressive disease (PD) biopsies were profiled by WES and 26 by WTS (Additional file 1: Table S1) [37, 38]. Twenty and 23 patients had paired BL and PD biopsies profiled by WES and WTS, respectively.

Patient clinical characteristics and tumor attributes are summarized (Table 1). The median follow-up duration was 20 months. The median age of patients was 45 years, and 77% of patients received palbociclib plus ET as first-line treatment. The median PFS was 15 months, and the disease progressed in 72% of patients, with 28% experiencing progression in < 6 months and 27% having a PFS duration of ≥ 20 months. PAM50 molecular classification of tumors indicated that 59 (50%) were luminal A subtype, 45 (38%) luminal B subtype, 7 (6%) HER2-enriched, 2 (2%) basal, and 5 (4%) normal-like (Additional file 1: Table S1). *BRCA1/2* pathogenic mutations were present in tumors in 12% of patients (Table 1).

**Table 1:** Summary of clinical and tumor attributes

	**Number**	**Median**	**Range**
Number of patients	71		
Age, years (median, range)	71	45	31–71
Menopausal status	70		
Premenopause	41 (59%)		
Postmenopause	29 (41%)		
Pathologic subtype	71		
IDC	67 (94%)		
ILC	2 (3%)		
Etc. and unknown	2 (3%)		
Breast cancer family history	53		
Yes	6 (11%)		
No	47 (89%)		
*BRCA1/2* pathogenic mutation^a^	64		
Wild-type	56 (88%)		
Mutated	8 (12%)		
Endocrine therapy	71		
Letrozole	48 (68%)		
Letrozole + GnRH agonist	2 (3%)		
Exemestane + GnRH agonist	14 (20%)		
Fulvestrant	7 (10%)		
Progressive disease event	51 (72%)		
PFS, months	71	15	95% CI (8.6, 20.4)
< 6	20 (28%)		
6–20	32 (45%)		
≥ 20	19 (27%)		
Metastasis	71		
De novo	32 (45%)		
Relapsed	39 (55%)		
RECIST tumor response	71		
Complete response	3 (4%)		
Partial response	29 (41%)		
Stable disease	27 (38%)		
Progressive disease	12 (17%)		
Palliative line of palbociclib	71		
First line	55 (77%)		
Second and later lines	16 (23%)		
Follow-up from palbociclib treatment (months)	71	20	3–48

*IDC* invasive ductal carcinoma, *ILC* invasive lobular carcinoma, *GnRH* gonadotropin-releasing hormone, *PFS* progression-free survival, *RECIST* Response Evaluation Criteria in Solid Tumors

^a^*BRCA1/2* pathogenic mutation was determined considering both germline and somatic mutations that truncate protein reading frame or annotated as pathogenic in ClinVar. Different types of endocrine therapies were combined with palbociclib. The median PFS of our cohort was 15 months, and the median follow-up duration was 20 months

Subgroup analyses of PFS according to patient and tumor clinical characteristics are shown in Fig. 1c, with corresponding associated statistics in Additional file 2: Table S2. A significant PFS benefit was observed in patients without previous neoadjuvant or adjuvant therapy compared with those who had received such treatment (hazard ratio [HR] = 2.58; *p* = 0.007, *q* = 0.075). Additionally, de novo stage IV disease was associated with significant benefits in PFS compared with relapsed metastatic disease (HR = 2.22; *p* = 0.009, *q* = 0.041).

### Tumor intrinsic molecular features associated with poor prognosis

Based on genomic and transcriptomic profiles of 96 tumors, we calculated a set of 36 molecular features representing different tumor intrinsic characteristics (Additional file 1: Table S1c). To characterize genomic instability resulting from DNA repair deficiencies such as HRD, we used somatic copy number alteration (CNA) patterns to quantify genomic scars on the basis of aneuploidy, loss of heterozygosity (LOH) [27], telomeric allelic imbalance (TAI), and large-scale transition (LST) [39]. Mutation signatures measure the contributions of distinct mutagenic processes to the somatic mutation catalogs of individual cancer genomes [31]. We identified 6 different mutation signatures in our cohort: S1, spontaneous mutational processes associated with age; S3, associated with homologous repair deficiency (HRD); S2 and S13, associated with the activity of the APOBEC family of cytidine deaminases; and S6 and S15, associated with defective DNA mismatch repair (COSMIC v2, https://cancer.sanger.ac.uk/signatures/). Also, we included numerical scores that represent mutation burden, chromosomal instability (CIN), tumor cell growth and proliferation, *BRCA1/2* pathogenic mutation status, and genomic alteration statuses of 11 known breast cancer genes (Additional file 1: Table S1c).

PFS in patients stratified by the molecular features of their BL tumors was compared to identify tumor intrinsic molecular markers that conferred worse prognosis (i.e., shorter PFS) (Fig. 1d, Additional file 6: Fig. S1, Additional file 2: Table S2). A high HRD index—an aggregate score of LOH, TAI, and LST—was associated with significantly shorter PFS (HR = 2.89; *p* = 0.001, *q* = 0.012) [27]. Mutation signature S3, which is associated with HRD and *BRCA1/2* mutations, was also associated with significantly shorter PFS (HR = 2.33; *p* = 0.016, *q* = 0.056). Likewise, the presence of mutated *BRCA1/2* was associated with shorter PFS (HR = 2.67; *p* = 0.012, *q* = 0.049). Mutation of *TP53* (HR = 3.92; *p* < 0.001, *q* < 0.001) and APOBEC signature S13 (HR = 3.19; *p* = 0.002, *q* = 0.012) were associated with shorter PFS. The luminal B tumor subtype is associated with more aggressive clinico-pathologic parameters than the luminal A subtype [40], and PFS was significantly shorter in patients with the luminal B subtype (*p* = 0.033, *q* = 0.088) and in patients with a high proliferative index (HR = 2.57; *p* = 0.005, *q* = 0.025) in univariate analysis. Multivariate analysis of the association of clinical and genomic tumor features with PFS revealed that *TP53* mutational status, tumor nuclear grade, and HRD signature 3 were independently associated with shorter PFS (Fig. 1e).

### Genomic feature analysis identified an HRD-high tumor cluster associated with poor prognosis

**Fig. 2:**
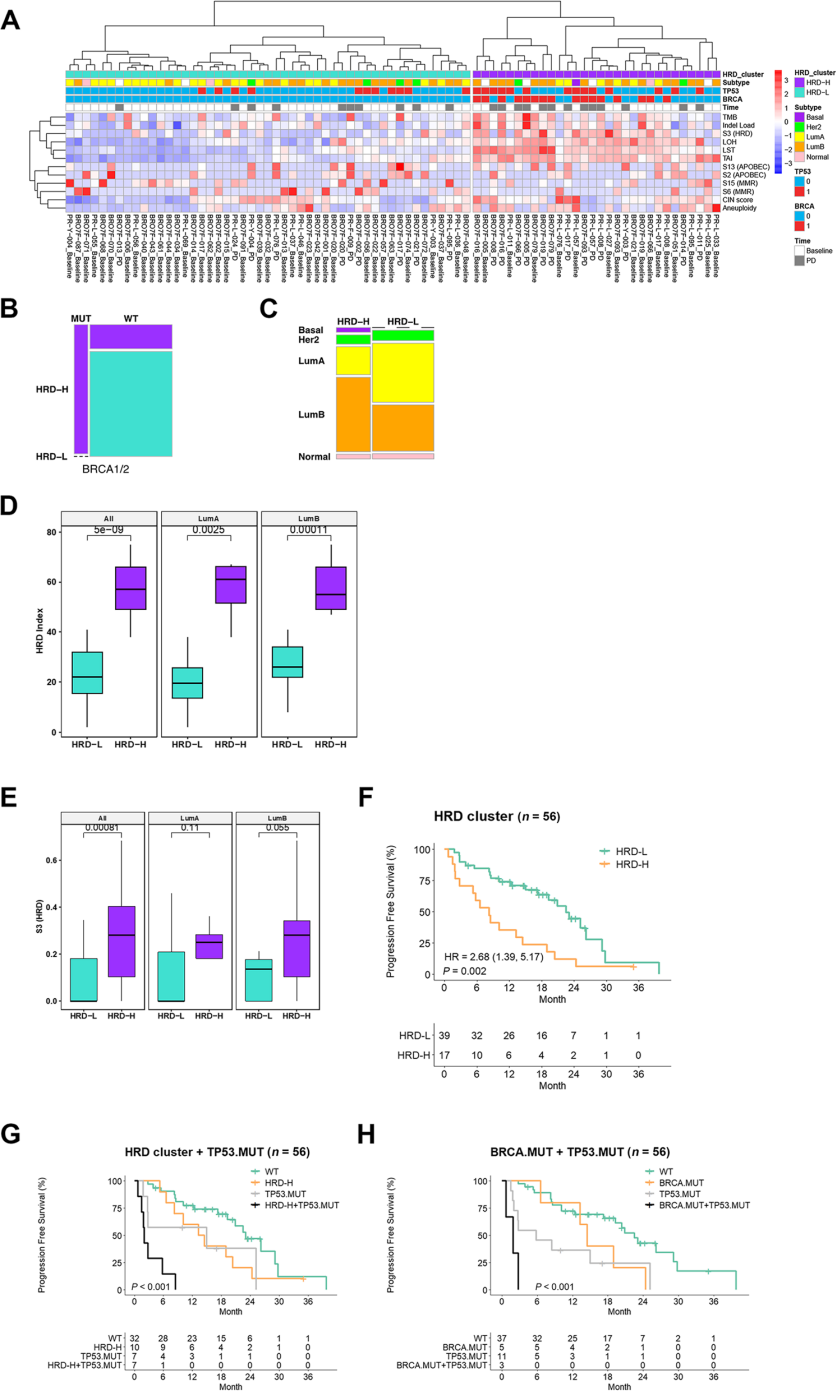
HRD-high tumors co-occurring with TP53 mutations are associated with worse PFS. **A** Unsupervised clustering of genomic features. HRD cluster: classification of tumors harboring higher (HRD-H) or lower (HRD-L) level of HRD genomic scar features. S2 and S13 (APOBEC): mutation signatures 2 and 13 linked to APOBEC enzyme activity. S3 (HRD): mutation signature linked to homologous recombination deficiency. CYT score: cytolytic activity score calculated from mRNA expression levels of *GZMA* and *PRF1*. CIN, chromosome instability; TAI, telomeraic allelic imbalance; LOH, loss-of-heterozygosity; LST, large-scale transitions. Subtype: PAM50 subtype classification. **B** HRD-H cluster significantly enriched in *BRCA1/2* mutations (Fisher’s exact test: *p* = 92.e−05). **C** HRD-H cluster significantly enriched in luminal B subtype (Fisher’s exact test: *p* = 0.0108436). **D**, **E** HRD-H cluster significantly enriched in HRD index (**D**) and mutation signature S3 (**E**) in the overall cohort and within luminal A and luminal B subtypes (Wilcoxon). **F** HRD-high cluster significantly associated with shorter PFS. **G** Kaplan-Meier plots comparing PFS between the four groups of baseline samples with different mutation statuses for *TP53* and *BRCA1/2*. HR and 95% CI shown in parentheses. BRCA.mut+TP53.mut: co-occurring *BRCA1/2* pathogenic mutation and *TP53* somatic mutations. **H** Kaplan-Meier plots comparing PFS between the four groups of baseline samples with different statuses of HRD cluster and *TP53* mutation. WT: *TP53* wild type and HRD-Low. TP53.mut: *TP53* mutation and HRD-Low. HRD-H: HRD-High and *TP53* wild type. HRD-H+TP53.mut: *TP53* somatic mutation and HRD-High

From the above analyses, we observed that multiple genomic markers linked to DNA damage and repair are associated with poor prognosis and tend to be correlated. To explore patient stratification based on these genomic features, we performed a hierarchical clustering of DNA-based genomic scar features, mutation signatures linked to defective DNA repair, and other genomic aberration features. Analysis revealed a distinct HRD-high cluster consisting of tumors from 33.9% of patients characterized by high levels of genomic scar features, HRD signature S3, and tumor mutation burden (Fig. 2a). HRD-high tumors were significantly associated with *BRCA1/2* mutations (*p* = 2e−05) and enriched in *TP53* mutations (*p* = 0.10; Fig. 2b, Additional file 6: Fig. S2a). HRD-high tumors were also associated with the luminal B subtype and a higher proliferative index in the overall cohort and with the HRD index within luminal A and B subtypes (Fig. 2c, d, Additional file 6: Fig. S2b). PD tumors were enriched in the HRD-high cluster, suggesting that this cluster is associated with disease progression (Additional file 6: Fig. S2c). Patients with BL HRD-high tumors had significantly shorter PFS compared with patients with HRD-low tumors (HR = 2.68; *p* = 0.002; *q* = 0.014; Fig. 2f).

*TP53* mutations are enriched in CDK4/6i-resistant HR+/HER2− MBC tumors, although *TP53* deficiency was not sufficient to promote CDK4/6i resistance in vitro [6]. We found that patients with BL tumors with *TP53* mutations and co-occurring HRD-high cluster had much shorter PFS compared with those with either *TP53* mutations in the absence of the HRD-high cluster, or patients with the HRD-high cluster in the absence of *TP53* mutations (*TP53* + HRD-high vs. wild type; HR = 16.3, *p* = 1.78e−07; *q* = 5.34e−07; Fig. 2g). Similarly, patients with co-occurring *TP53* and *BRCA1/2* mutations had much shorter PFS compared with those who had either *TP53* mutations or *BRCA1/2* mutations alone (*TP53* + *BRCA1/2* vs. wild type; HR = 31.6, *p* = 4.96e−06; *q* = 1.49e−05 Fig. 2h). These findings suggest that combined defective homologous recombination (HRD-high cluster or *BRCA1/2* mutation) and impaired *TP53* may denote a highly resistant tumor phenotype. HRD-high tumors with co-occurring *TP53* mutations were significantly associated with a higher HRD index, higher proliferative index, and decreased expression of *CDKN1A*, a regulator of cell cycle arrest in response to DNA damage (Additional file 6: Fig. S3a, 3b, 3d) [41]. HRD-high tumors with co-occurring *TP53* mutations were also significantly associated with decreased expression of the estrogen early response gene set (Additional file 6: Fig. S3c). Taken together, these results suggest that HRD-high cluster/*TP53-*mutant tumors have gained a proliferative advantage with dysregulated response to DNA damage and diminished dependence on estrogen signaling for growth, which conferred intrinsic resistance to palbociclib plus ET.

### Gene expression analysis identified a proliferative tumor cluster with poor prognosis

Because palbociclib inhibits cell cycle progression, we examined gene expression signatures and patterns of cell cycle regulatory genes on BL tumors to identify genes potentially contributing to resistance. Among HR+ breast cancer patients who progressed on ET, high *CCNE1* expression was previously shown to be associated with lower efficacy of palbociclib plus fulvestrant [11]. In the current cohort of BC patients, most of whom had not received prior aromatase inhibitor therapy, *CCNE1* expression was not associated with decreased PFS, although a trend toward shorter PFS was seen in patients expressing higher *CCNE1* and *CCNE2* levels (Additional file 6: Fig. S4a, 4b). High expression of the cell cycle regulatory transcription factor *E2F1* was significantly associated with shorter PFS (HR = 2.0; *p* = 0.021; *q* = 0.062; Additional file 6: Fig. S4c). The multigene analysis increases sensitivity in identifying trends and associations that may be too weak at the individual gene level. We performed a gene signature analysis on 935 gene sets consisting of cancer hallmark and canonical signaling pathways and identified high expression of cell cycle regulatory sets, *E2F* and *RB1* targets, as significantly associated with shorter PFS (Additional file 6: Fig. S4d, 4e, Additional file 3: Table S3). The mechanistic target of rapamycin 1 (mTORC1) signaling has been implicated as a mechanism of escape in ER-positive MBC [42]. We found that high expression of the *mTORC1* signaling gene set was also significantly associated with shorter PFS (Additional file 6: Fig. S4f). Low expression of progesterone receptor (PGR) in estrogen receptor (ER)-positive tumors is associated with more aggressive and proliferative disease [43]. In our cohort, tumors expressing low levels of the PGR or of the estrogen early response gene set exhibited markedly shorter PFS compared with tumors expressing high levels of these markers (Additional file 6: Fig. S4g, 4h), suggesting that palbociclib plus ET is less effective in the subpopulation of HR+/HER2− MBC that bypassed reliance on hormone signaling for growth.

To examine the correlation and stratify patients based on the various gene expression-based poor prognosis markers, we performed an unsupervised clustering of 30 gene expression signatures with a significant prognostic association in BL and PD tumors and identified a proliferative cluster (PC2) of tumors enriched in cell cycle expression signatures (Additional file 6: Fig. S5a). PC2 was significantly associated with a higher proliferative index, HRD index, HRD signature S3, and enriched in *BRCA1/2* and *TP53* mutations, compared with the low proliferative cluster (PC1) (Additional file 6: Fig. S5b‒g). Although PC2 was enriched in luminal B and HER2-enriched subtypes (Additional file 6: Fig. S5e), it remained associated with a higher proliferative index within both luminal B and luminal A subtypes (Additional file 6: Fig. S5b). Patients with PC2 had shorter PFS than patients with PC1 (HR = 3.11, *p* < 0.001; *q* = 0.002; Additional file 6: Fig. Si). Together, these findings suggest that tumor proliferation, reflected by higher expression of multiple cell cycle regulatory genes and pathways, could be a hallmark of drug-resistant and rapidly progressing tumors.

### Integrative analysis of molecular features identified distinct prognostic tumor clusters

Multi-omics tumor profiles provide a unique opportunity to discover novel molecular subtypes of HR+ MBC in the context of palbociclib plus ET resistance. To further stratify patients within poor prognosis groups characterized by higher levels of HRD or tumor proliferation, we performed an integrative clustering analysis of genomic and gene expression-based features with significant PFS association and identified four distinct clusters, termed IC1–IC4 (Additional file 6: Fig. S6a). Compared with patients with IC1 tumors, patients with IC2‒IC4 tumors had significantly shorter PFS (Additional file 6: Fig. S6b). The poor prognosis clusters IC2‒IC4 were highly enriched in the luminal B subtype, the high proliferation cluster PC2, the HRD-high cluster, and *BRCA1/2* mutations, compared with IC1 (Additional file 6: Fig. S7a-c). The favorable prognosis cluster IC1 had a lower proliferative index and a higher estrogen response signature than poor prognosis clusters (Additional file 6: Fig. S7e, 7f). The poor prognosis clusters IC2‒IC4 were differentiated by distinct molecular features. Compared with clusters IC1, IC3, and IC4, IC2 had a high APOBEC signature 13 score (Additional file 6: Fig. S7g) and increased prevalence in PD relative to BL tumors (Additional file 6: Fig. S7d). IC3 is the largest of the poor prognostic clusters with the highest proliferative index (Additional file 6: Fig. S7e). Cluster IC4 had the highest HRD signature S3 score (Additional file 6: Fig. S7h) and lowest estrogen response signature (Additional file 6: Fig. S7f). These findings suggest that the integrative approach to examining multiple types of molecular markers could be more effective than the univariate approach in delineating a complex landscape of molecular mechanisms driving drug resistance and poor prognosis in HR+/HER2− MBC.

### Post-treatment changes in tumor molecular profiles

**Fig. 3:**
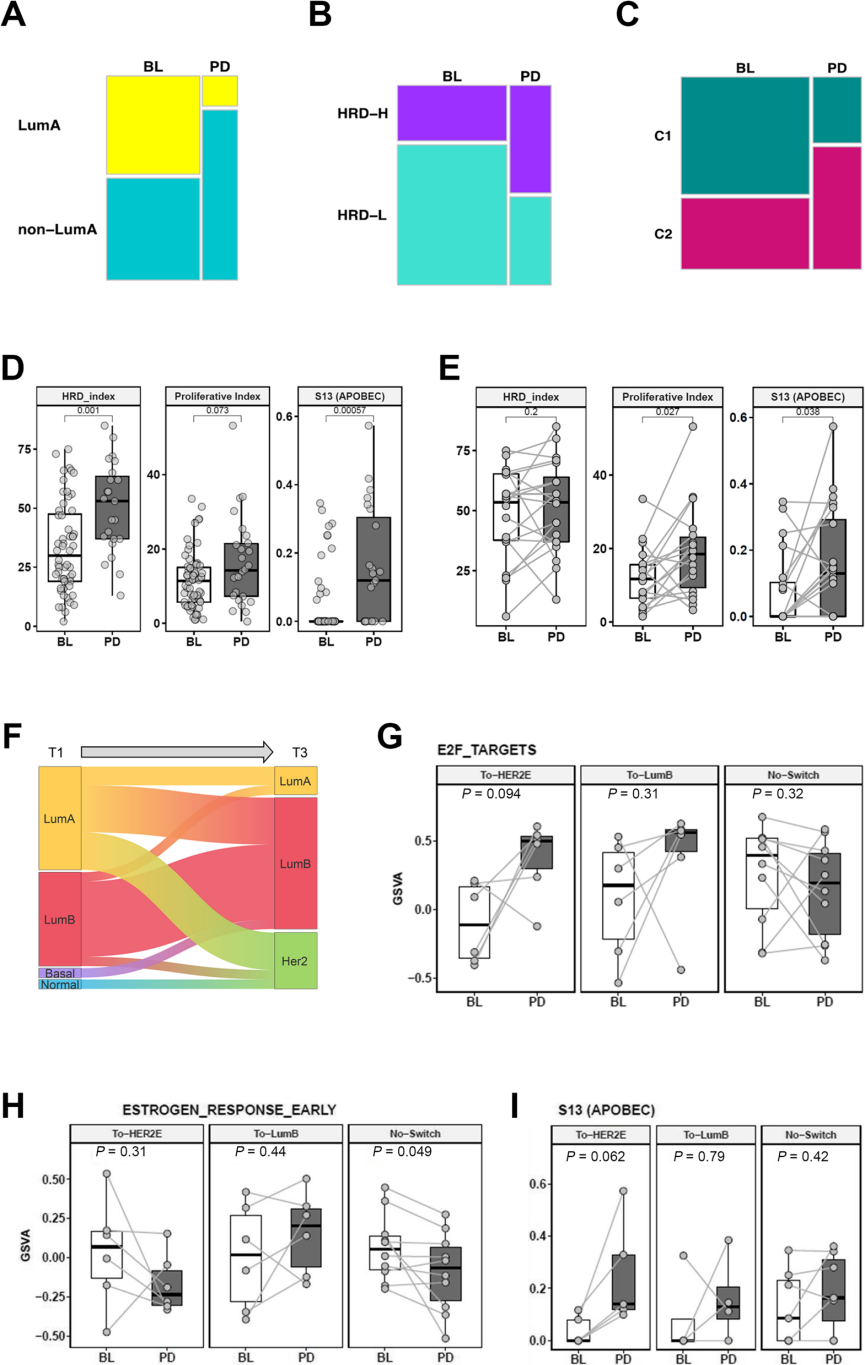
Post-treatment enrichment of resistance markers. The non-luminal A subtype (**A**), HRD-H cluster (**B**), and proliferative cluster (**C**) were enriched in PD compared to baseline (Fisher’s exact test: *p* = 0.0084 for subtype; *p* = 0.054 for HRD cluster; *p* = 0.035 for proliferative cluster). Comparing the changes in HRD index, proliferative index, and S13 mutation signature at BL vs. PD among all samples (**D**) and longitudinally paired samples (**E**). The non-luminal A subtype (**A**), HRD-H cluster (**B**), and proliferative cluster (**C**) were enriched in PD compared to baseline (Fisher’s exact test: *p* = 0.0084 for subtype; *p* = 0.00314 for HRD cluster; *p* = 0.06351 for proliferative cluster). Comparing the changes in HRD index, proliferative index, and S13 mutation signature at BL vs. PD among all samples (**D**) and longitudinally paired samples (**E**). **F** Sankey diagram showing the switching of subtypes from luminal A at BL into HER2E or luminal B subtypes at PD. Comparing the changes in E2F targets signature (**G**), estrogen response early signature (**H**), and S13 mutation signature (**I**) between paired BL and PD tumors among the three groups of patients. To-HER2E: subtypes switched to HER2E at PD. To-LumB: subtypes switched to luminal B at PD. No-Switch: subtypes remained the same between BL and PD. GSVA: signature scored calculated by gene set variation analysis

To characterize tumor intrinsic molecular changes during palbociclib plus ET treatment, we compared molecular profiles of BL and PD tumors and observed that poor prognosis markers were enriched in PD tumors relative to BL tumors. These enriched markers included non-luminal A subtypes, the HRD-high cluster and HRD index, the proliferative cluster PC2 along with the proliferative index, and APOBEC mutation signature S13 (Fig. 3a‒d). To investigate the causes of these enrichment patterns, we further examined the longitudinal changes that only occurred in BL/PD paired samples (Fig 3e–i). While the HRD index was higher in PD samples overall, it did not increase from BL to PD in the paired tumors, suggesting post-treatment enrichment of the HRD index might arise from selection bias in the PD sample group toward patients with poor prognosis (Fig. 3d, e). On the other hand, in longitudinally paired BL and PD tumors, the proliferative index and the APOBEC signature S13 were both significantly higher in tumors post-progression (*p* = 0.027 and *p* = 0.038, respectively; Fig. 3e), indicating selective pressure for APOBEC activation and tumor cell proliferation during treatment.

The PAM50 molecular subtype in breast cancer can provide important prognostic information and has been shown to switch post-chemotherapy treatment [44]. Among longitudinal pairs, we also observed frequent switching of PAM50 molecular subtypes from BL to PD. Tumors that were luminal A pretreatment frequently switched into luminal B or HER2-enriched subtypes at PD (Fig. 3f), reflecting the strong increases in PAM50 subtype scores for luminal B and HER2E (Additional file 6: Fig. S8a, 8b). Subtype switching was also accompanied by changes in distinct molecular signatures. E2F expression target signatures increased from BL to PD only in tumors switching into luminal B or HER2-enriched subtypes (Fig. 3g). Tumors switching to the HER2-enriched subtype had a diminished ER signaling signature, whereas tumors switching to the luminal B subtype tended to retain ER signaling (Fig. 3h). The APOBEC score was also significantly higher in tumors switching into the HER2-enriched subtype (Fig. 3i). Hence, post-treatment enrichment of poor prognosis markers may be part of a treatment-induced transcriptomic rewiring that drives cancer cell proliferation.

Increased tumor proliferation post-treatment is also revealed through markedly higher individual cell cycle regulatory gene expression from BL to PD tumors. Two key cell cycle regulatory genes, *CCNE1* and *CCNE2*, which are implicated in CDK4/6i plus ET resistance [6, 11], as well as *E2F1* [2], the key downstream target of *RB1*, were significantly increased in PD versus BL tumors (Additional file 6: Fig. S9a, 9b) [2]. *CCNE1* and *CCNE2* expression only increased in tumors that switched subtypes (Additional file 6: Fig. S9c, 9d). To confirm post-treatment upregulation of cell cycle regulatory genes, IHC analysis was performed. As illustrated by a patient biopsied at BL and post-progression, more intense staining of cyclin E1 and cyclin E2 was seen in the PD tumor cells with higher Ki67 and higher phosphorylated retinoblastoma protein levels compared with cells from BL (Additional file 6: Fig. S10a), consistent with the corresponding gene expression patterns in the cohort and in paired BL and PD tumors (Additional file 6: Fig. S10b, 10c).

### Acquired genomic alterations potentially confer drug resistance

Genomic profiling and comparative analysis of matched pre- and post-treatment samples allow a dissection of which genetic alterations are acquired during treatment and confer drug resistance.

**Fig. 4:**
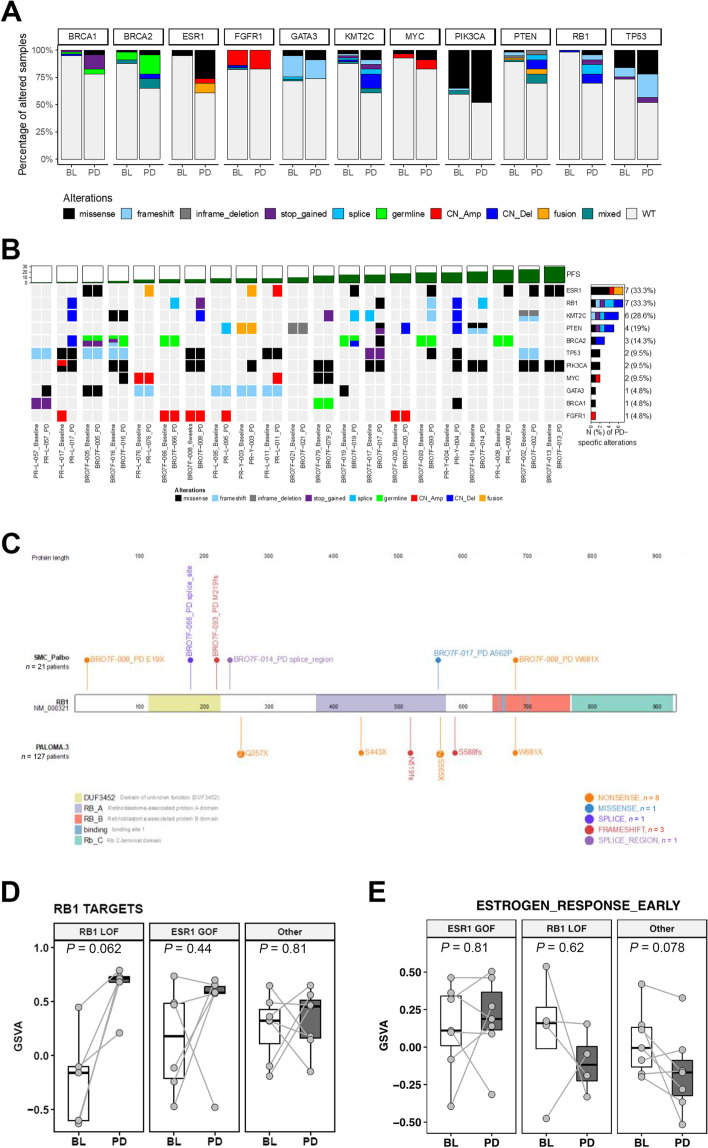
Landscape of PD-specific genomic alterations. **A** Stacked bar plots comparing the prevalence of genomic alterations at BL vs. PD for key BC genes. Colors represent the different genomic alterations including WT, missense, frameshift, inframe deletion, nonsense (splice site mutation, germline mutation) copy number amplification, copy number deletion, fusion, and mixed, indicating the tumor harbored multiple alterations. **B** Oncoprint of the mutational profile of selected genes for patients with paired baseline/on-treatment and PD samples. Paired samples for the same patient grouped together to highlight PD-specific alterations. Patients ordered by PFS as indicated by the track above the oncoprint. Colors represent the different mutation types. Stacked bar plots and number on the right show the number of patients with PD-specific alterations, with the percentage in parenthesis indicating the frequency of PD-specific alternations in 21 patients. **C** PD-specific RB1 mutations from our cohort in comparison with the spectrum of RB1 mutations reported in the PALOMA-3 study. We observed 6 mutations from 23.8% (5/21) of patients. Comparing longitudinal changes in RB1 TARGETS (**D**) and ESTROGEN RESPONSE EARLY signatures (**E**) between paired BL and PD tumors among the three groups. RB1 LOF: patients harboring PD-specific RB1 loss-of-function genomic alterations at PD. ESR1 GOF: patients harboring PD-specific ESR1 gain-of-function genomic alterations at PD. Other: all other patients with paired BL and PD samples. Patient BRO7F-093 harbored acquired mutations in both RB1 and ESR1 and was included in the “RB1 LOF” group in **D** and “ESR1 GOF” group in **E**. RB1 TARGETS: EGUCHI CELL CYCLE RB1 TARGETS gene set from mSigDB v5.2

Multiregion sequencing has revealed intra-tumoral and spatial heterogeneity of mutations in breast cancers [45], raising the caveat that differences between genomic profiles of paired tumor biopsies are not necessarily acquired or functionally relevant. To mitigate this issue, we focused on eleven breast cancer-associated genes (Additional file 4: Table S4) that are likely to be functional based on prior publications and that have frequent genomic alterations in our cohort and examined individual genomic alterations and associated changes in expression signatures. A comparison of BL and PD tumors revealed significantly increased frequencies of somatic genomic alterations in PD tumors for six BC-associated genes, *BRCA1* (*p* = 0.040), *BRCA2* (*p* = 0.028), *ESR1* (*p* = 0.00043), *KMT2C* (*p* = 0.012), *PTEN* (*p* = 0.044), and *RB1* (*p* = 0.0005) (Fig. 4a; Additional file 4: Table S4). However, post-treatment enrichment of poor prognosis markers such as THE HRD index may arise from a selection bias toward patients who are poor responders. To enrich for genomic alterations acquired because of selective pressure from treatment, we defined PD-specific genomic alterations as somatic mutations, copy number amplifications and deletions, and gene fusions that were only detected in PD but not in paired BL or on-treatment tumors. *ESR1* and *PIK3CA* are commonly mutated in HR+/HER2− MBC, although *ESR1* mutations are only present after ET whereas *PIK3CA* mutations are also observed before treatment [2, 5, 6, 46]. In paired BL and PD tumors in our cohort, 33% of paired PD samples acquired *ESR1* alterations, whereas 9.5% acquired *PIK3CA* mutations (Fig. 4b). Most of the specific acquired mutations in *ESR1* identified in our PD samples overlapped with mutations acquired in patients from the PALOMA-3 trial who were treated with endocrine monotherapy or palbociclib plus fulvestrant [5, 47] and appear to be gain-of-function alterations (Additional file 6: Fig. S11a).

A relatively high level of acquired alterations in *RB1* (33%), the histone-lysine methyltransferase *KMT2C* (29%), and *PTEN* (19%) were also identified in the PD tumor samples, suggesting a role in driving resistance to therapy in this patient cohort (Fig. 4b). *RB1* loss-of-function alterations are known to confer resistance to CDK4/6i plus ET, but acquired events were previously reported only in a minority of patients, typically less than 10% [4–6]. Five of the six *RB1* mutations observed in our cohort are heterogeneous patterns of loss-of-function alterations such as nonsense or frameshift and affected 19% (4/21) of paired PD samples, suggesting a greater prevalence of cell cycle deregulation as an acquired resistance mechanism than previously reported (Fig. 4c). *KMT2C* mutations are common in MBC [48], although the acquisition of genomic alterations in this gene has never been reported in patients treated with CDK4/6i. We found a relatively high level (28.6%) of acquired alterations in patients in this study, with the majority of alterations consistent with loss-of-function and therefore suggestive of a tumor-suppressor role (Additional file 6: Fig. S11b). *PTEN* loss-of-function alterations confer resistance to CDK4/6i plus ET and have recently been identified in post-progression tumors in 2/5 patients treated with ribociclib plus ET [49]. Our observed high rate of *PTEN* alterations is consistent with this previous report and suggests a role for *PTEN* loss in driving CDK4/6i plus ET resistance.

Post-progression tumors acquiring *RB1* alterations were associated with higher expression of *RB1* targets and lower estrogen response signature, indicating cell cycle progression with diminished dependence on estrogen signaling (Fig. 4d, e). APOBEC mutation signature S13 was a marker of poor prognosis at baseline (Fig. 1d) that significantly increased between paired BL and PD samples (Fig. 3e). Notably, increased S13 at PD was associated with tumors harboring acquired loss-of-function *RB1* (Additional file 6: Fig. S12a), which also exhibited stronger upregulation of replication stress signature, *APOBEC3B* and *CHEK1* expression than other PD tumors (Additional file 6: Fig. S12b-d). It has been reported that oncogene, tumor suppressor, and drug-induced DNA replication stress induce *APOBEC3B*-mediated mutagenesis in BC [50]. Tumor clonal evolution analysis performed on paired BL and PD tumors revealed that the APOBEC signature S13 is enriched in PD-specific tumor subclones that emerged post-treatment (Additional file 6: Fig. S13a). In 1 patient, the APOBEC mutation signature S13 was not detectable in the main BL subclone C2 but was the predominant fraction in subclone C3, which emerged with an acquired *RB1* A562P mutation and became the major subclone at PD (Additional file 6: Fig. S13b-d). These results suggest that during tumor clonal evolution, palbociclib plus ET treatment selected for tumor subclones with *RB1* loss-of-function that mediated cell cycle deregulation and potentially led to elevated levels of replication stress and the subsequent induction of APOBEC-mediated mutagenesis.

## Discussion

Resistance to CDK4/6i plus ET is an important unmet medical need, yet there are no clinically viable biomarkers that can distinguish patients who progress earlier than expected, or therapeutic targets that can be exploited to overcome resistance [2]. Here, we performed, to our knowledge, the first prospectively enrolled comprehensive multi-omics analysis of baseline and post-progression tumors from patients receiving palbociclib in combination with ET. Ours is also the first study employing an integrative, multi-omics approach to stratify patients and delineate the heterogeneous molecular mechanisms contributing to poor prognosis and CDK4/6i resistance. We have identified novel molecular features and integrative subtypes associated with poor prognosis and novel, as well as previously identified alterations, in genes that potentially drive acquired resistance. Because this was a single-arm study, we can enrich for baseline molecular features that mediate CDK4/6i plus ET resistance but cannot differentiate these from intrinsic prognostic factors. Molecular differences observed between paired biopsies taken pre- and post-treatment provide strong evidence for acquired drug resistance mechanisms but could also be explained by factors such as tumor heterogeneity. While still in need of validation in larger and independent cohorts, the initial findings shed new light on molecular mechanisms of CDK4/6i plus ET resistance and therapeutic strategies that could overcome it. Although the type of endocrine therapy AI vs. fulvestrant is a potential confounder, its effects may be minor as 64 patients (90%) were treated with AIs.

Comparing molecular profiles of paired BL and PD samples, we observed a high frequency of tumor molecular subtype switching at progression, particularly from luminal A into more aggressive luminal B and HER2-enriched subtypes. Tumors switching to HER2-enriched were characterized by an increased APOBEC signature, expression of cell cycle regulatory genes, and a tendency toward the decreased expression of estrogen-response genes. Interestingly, tumors switching to luminal B retained or increased expression of estrogen response genes. These findings demonstrate the high plasticity of tumors under selective pressure from palbociclib plus ET, which could drive resistance through the dysregulation of multiple pathways such as cell cycle, hormone receptor, and growth factor receptor–mediated signaling [51].

Acquired alterations in several cell cycle regulators and BC-associated oncogenic genes in response to CDK4/6i therapy have been reported [2, 6]. We also observed increased frequencies of genomic alterations at PD for BC genes such as *BRCA1/2* and *TP53* and notably a high prevalence of acquired alterations affecting *ESR1*, *RB1*, *KMT2C*, and *PTEN*. Mutations affecting the histone methyltransferase gene *KMT2C* are frequent in ER-positive BC, and *KMT2C* loss has been associated with a poor prognosis in patients on ET [48]. We observed frequent loss-of-function alterations in *KMT2C* in post-progression tumors. It is possible that these mutations are driven by the ET component of therapy, resulting in a diminished response to ET. Further study is needed to determine if loss of *KMT2C* can confer resistance to CDK4/6i plus ET specifically. Paired ctDNA sequencing of 195 patients from PALOMA-3 has revealed frequent acquisition of *ESR1* mutations in both the fulvestrant-only and the palbociclib plus fulvestrant treatment arms, implicating *ESR1* gain-of-function in the development of acquired resistance [5]. Our finding that 33% (7/21) of paired PD samples harbor acquired *ESR1* alterations, most in known mutation hotspots [47], strengthens the argument that endocrine resistance is a major driver of resistance to combination therapy. A recent report identified attenuated ER activity and expression in TP53 mutant tumors and tumors with simultaneous presence of ESR1 and TP53 mutation also showed reduced ER activity [52]; two cases with co-occurrences of TP53 and ESR1 mutations were associated with reduced ER activity in our cohort. In addition, it has been reported that mutations in TP53 and ESR1 are mutually exclusive. However, we did not find a significant association between TP53 and ESR mutation (data not shown), which might be due to the small number of ESR1 mutated patients in our cohort.

Preclinical and clinical studies have demonstrated that *RB1* loss confers CDK4/6i plus ET resistance [5, 6]; however, clinical studies have found loss-of-function *RB1* alterations only in a small fraction of patients. The PALOMA-3 trial revealed that *RB1* mutations emerged only in patients receiving palbociclib plus fulvestrant and in just 4.7% of those patients [5]. WES of 59 tumors with CDK4/6i exposure found *RB1* loss-of-function alterations occurred in 9.8% of resistant tumors [6]. We also found the acquisition of *RB1* alterations in PD tumors, most of which were likely to be loss-of-function. However, acquired *RB1* LOF mutations affected 19% of paired PD tumors in this cohort, suggesting a higher prevalence of acquired *RB1* mutations in the PD patient population than previously reported. This result remains to be independently replicated by additional clinical studies with larger cohorts. Increased prevalence of mutations in *BRCA1/2* and *TP53* have been reported in primary tumors from Asian patients with breast cancer [53]. *RB1* mutations and *KMT2C* mutations may be more prevalent in Asian populations, although the line of therapy or endocrine partner may also contribute. Further study and validation in an independent prospective cohort are warranted.

The HRD signature reflects functional defects in the homologous recombination repair pathway, of which the best-known cases are germline mutations disrupting BRCA1/2 function. In our cohort, measures of HRD by the mutational signature, S3 or HRD index, an aggregate score of genomic scars caused by homologous recombination deficiency, were each significantly associated with shorter PFS. Further analysis revealed a distinct cluster of tumors, designated HRD-high, that were highly enriched in genomic scar features linked to HRD and associated with poor prognosis. The HRD-high cluster consisted of tumors from 34% of patients, whereas only 12% harbored *BRCA1/2* loss-of-function mutations. The HRD phenotype renders tumors sensitive to agents, such as poly (ADP-ribose) polymerase (PARP) inhibitors, that induce DNA double-strand breaks [54]. Our findings suggest that a unique cluster of resistant tumors, including but not limited to those harboring *BRCA1/2* loss-of-function mutations, could be sensitive to DNA double-strand-break-inducing agents.

*TP53* is known as the “guardian of the genome” and plays a key tumor suppressor role in the regulation of cell cycle progression, apoptosis, and genomic stability [55]. Mutant or deficient *TP53* was previously found to be insufficient to promote CDK4/6i resistance in vitro [6]. However, because of previously observed increases in *TP53* alterations in CDK4/6i resistant biopsies, which we also observed in PD tumors, it was suggested that the *TP53* mutation may be permissive for the development of other resistance-promoting alterations or may cooperate with additional alterations to mediate resistance in vivo [6, 51]. In support of this, we found that patients with co-occurring BL mutant *TP53* and HRD-high exhibited a highly proliferative phenotype that was independent of estrogen signaling, with a markedly poor prognosis. The combined mutant *TP53*/HRD-high cluster could be a novel biomarker to identify patients who respond poorly to CDK4/6i and who may benefit from PARP inhibitors or other DNA double-strand-break-inducing agents. Furthermore, PARP inhibitors may overcome endocrine resistance in patients with ER-positive MBCs who harbor germline BRCA1 and/or 2 mutations or HRD features. Thus, we proposed a clinical trial evaluating talazoparib activity in this clinical setting, and currently, this clinical trial is ongoing (NCT04819243).

In our BL cohort, the APOBEC signature S13 was significantly associated with shorter PFS at BL and markedly increased prevalence in PD tumors. Our findings extend previous reports that the APOBEC signature was induced in resistant tumors from two patients receiving palbociclib plus fulvestrant [5] and that APOBEC-mediated mutagenesis in response to targeted therapy treatment may fuel the development of acquired resistance in non–small cell lung cancer [56]. Furthermore, our comparison of paired BL and PD samples revealed that increased APOBEC signature S13 was associated with subtype switching to HER2-enriched, acquired *RB1* alterations and upregulation of DNA replication stress markers, suggesting that APOBEC-mediated mutagenesis may be a consequence of acquired resistance mechanisms that counter CDK4/6i mediated cell cycle arrest. Replication stress is a cancer-specific vulnerability that can be exploited through inhibition of S-phase checkpoint kinases such as ATR and CHK1 among other therapeutic agents that can enhance replication stress and push cancer cells toward mitotic catastrophe [57].

Our multi-dimensional analysis of CDK4/6i plus ET resistance mechanisms revealed a complex interplay between two major cancer hallmarks: unchecked cell proliferation versus genomic instability and DNA damage. Tumor proliferation, characterized by cell cycle regulatory gene expression, expression signatures, or IHC analysis, was associated with poor prognosis at baseline and markedly increased post-treatment. DNA damage, characterized by somatic mutation signatures attributed to HRD and APOBEC as well as CNA-based genomic scar features such as HRD index, was associated with poor prognosis at baseline and acquired resistance at PD, likely enriched through treatment-induced selective pressure and tumor clonal evolution. The two hallmarks are correlated: BL tumors in the proliferative cluster PC2 were enriched in the HRD-high cluster, and both proliferative index and APOBEC mutation signatures markedly increased post-treatment. On the one hand, breast cancers evolved resistance mechanisms, such as *RB1* inactivation or *ESR1* gain-of-function changes, to deregulate cell cycle control and drive aggressive cell growth and proliferation. On the other hand, unchecked cell growth and proliferation lead to replicative stress or DNA repair deficiencies that accumulate DNA damage, exacerbate genomic instability, and trigger programmed cell death. That may be why HRD-high tumors with co-occurring *TP53* mutations were associated with very poor prognosis because *TP53* loss-of-function would impair DNA damage response and apoptosis/senescence signaling, thereby making the cells more resistant to cell cycle arrest and more tolerant of increased DNA damage at the same time. While HRD and APOBEC-mediated mutagenesis could fuel oncogenesis and drug resistance, these consequences of aberrant cell proliferation could also give rise to tumor-specific vulnerabilities that can be therapeutically targeted.

## Conclusions

We identified novel molecular features and integrative subtypes associated with poor prognosis and gene alterations that potentially drive acquired resistance. Our findings will help identify patients who will benefit most from treatment with CDK4/6i plus endocrine therapy and find therapeutic targets to overcome resistance, intrinsic and acquired, that may improve the treatment of HR+/HER2− metastatic breast cancer.

## Supplementary Information

**Additional file 1. Table S1.** Sample-levelannotation including patient clinical attributes, tumor characteristics andgenomic/molecular features.

**Additional file 2. Table S2. **PFS association statistics for clinical variables and molecular features e.g. p-value, HR ratio, median PFS.

**Additional file 3. Table S3. **PFS associationstatistics for gene signatures (GSVA scores).

**Additional file 4. Table S4. **Comparison of genomic alteration prevalence at BL vs. PD.

**Additional file 5. Table S5. **Summary of oncogenic events in paired PDtumors. Endocrine therapy: endocrine therapy in combination with Palbo treatment.

**Additional file 6. Fig. S1. **Kaplan-Meier plots of poor prognostic biomarkers. **Fig. S2.** Characteristics of the HRD-high cluster. **Fig. S3.** Characteristics of HRD-high tumors co-occurring with TP53 mutation. **Fig. S4.** Kaplan-Meier plots of expression-based prognosis markers. **Fig. S5.** Proliferative cluster enriched in poor prognostic markers. **Fig. S6.** Integrative analysis identified distinct prognostic clusters. **Fig. S7.** Molecular characteristics of integrative clusters. **Fig. S8.** Subtype switching driven by changes in PAM50 score composition. **Fig. S9.** Increased tumor growth and proliferation at PD. **Fig. S10.** IHC analysis of cell cycle markers. **Fig. S11.** Landscape of PD-specific genomic alterations. **Fig. S12.** RB1 LOF associated with increased APOBEC signature at PD.** Fig. S13. **APOBEC signature enriched in PD-specific tumor subclones.

## Data Availability

Raw sequencing reads are available from EGA EGAS00001005736 (https://ega-archive.org/studies/EGAS00001005736) for WES and WTS [37]. Gene expression values (TPM) are available from NCBI GEO GSE186901 (https://www.ncbi.nlm.nih.gov/geo/query/acc.cgi?acc=GSE186901) [38]. The raw data is available under controlled access according to the institution’s policy for personal data protection. Data access can be obtained by contacting Prof. Yeon Hee Park [yhparkhmo@skku.edu]. The remaining data are available within the article and supplementary information or available from the authors upon request. The source data are provided in this paper.
