# Supplementary material for: Longitudinal multi-omics study of palbociclib resistance in HR-positive/HER2-negative metastatic breast cancer
Source: Genome Med. 2023 Jul 20;15:55. doi: 10.1186/s13073-023-01201-7 (PMC10360358; doi:10.1186/s13073-023-01201-7)
Supplement: Supplementary file 6 — Additional file 6. Fig. S1. Kaplan-Meier plots of poor prognostic biomarkers. Fig. S2. Characteristics of the HRD-high cluster. Fig. S3. Characteristics of HRD-high tumors co-occurring with TP53 mutation. Fig. S4. Kaplan-Meier plots of expression-based prognosis markers. Fig. S5. Proliferative cluster enriched in poor prognostic markers. Fig. S6. Integrative analysis identified distinct prognostic clusters. Fig. S7. Molecular characteristics of integrative clusters. Fig. S8. Subtype switching driven by changes in PAM50 score composition. Fig. S9. Increased tumor growth and proliferation at PD. Fig. S10. IHC analysis of cell cycle markers. Fig. S11. Landscape of PD-specific genomic alterations. Fig. S12. RB1 LOF associated with increased APOBEC signature at PD. Fig. S13. APOBEC signature enriched in PD-specific tumor subclones. [file 13073_2023_1201_MOESM6_ESM.docx]

F**Figure S1. Kaplan-Meier plots of poor prognostic biomarkers**

Kaplier-Meier plots of genomic markers significantly associated with shorter PFS – HRD index (A), PAM50 subtype (B), BRCA1/2 pathogenic mutation status (C), TP53 somatic mutation status (D), mutation signatures S3 (E) and S13 (F), tumor mutation burden (TMB) (G) and proliferative index (H). APOBEC: apolipoprotein B editing complex. CI: Confidence Interval. HRD: Homologous Recombination Deficiency. Signature: COSMIC mutational signature. WT: wild type. MUT: mutation. PAM50: intrinsic breast cancer subtype. LumA: Luminal A. LumB: Luminal B. Non-Luminal: HER2-enriched, basal or normal-like PAM50 subtype. TMB: Tumor Mutation Burden. BRCA status: BRCA1/2 pathogenic mutations. HRD index was split by median into high and low groups.


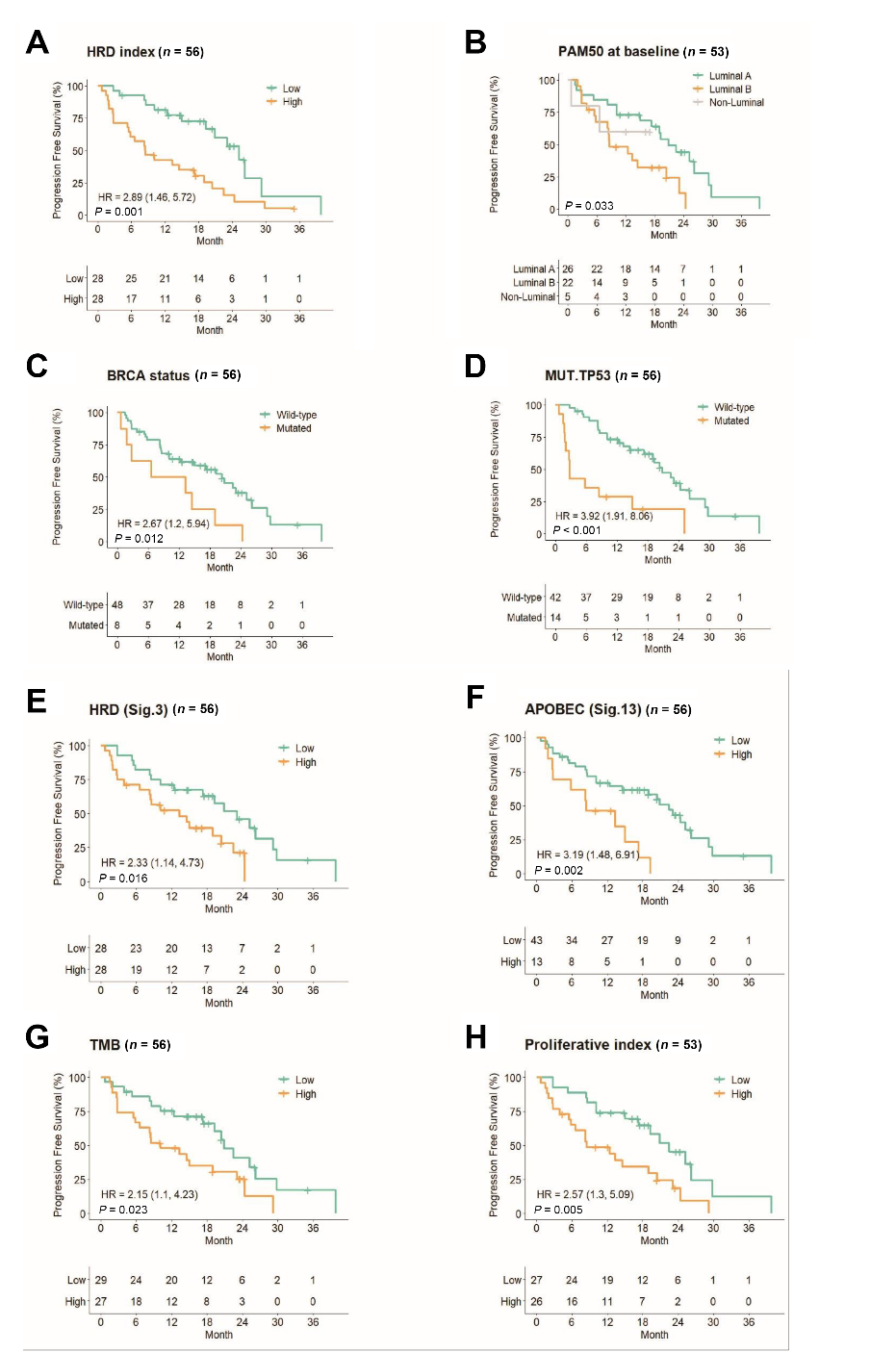


**Figure S2. Characteristics of the HRD-high cluster**

HRD-H cluster is enriched in *BRCA1/2* pathogenic mutations (A) (Fisher’s exact test: *p*=2e-05), TP53 mutations (B) (Fisher’s exact test: *p*=0.10486), PD vs. BL (C) (Fisher’s exact test: *p*=0.00276) and in luminal B subtype (D) (Fisher’s exact test: *p*=0.036). HRD-H cluster is significantly enriched in the HRD index in the overall cohort and within luminal A and luminal B subtypes (E) (Wilcoxon rank sum test). HRD-H cluster is significantly enriched in the mutation signature S3 in the overall cohort (F) (Wilcoxon rank sum test). Proliferative index is significantly enriched in the HRD-H cluster in the overall cohort (G) (Wilcoxon rank sum test). HRD: Homologous Recombination Deficiency.


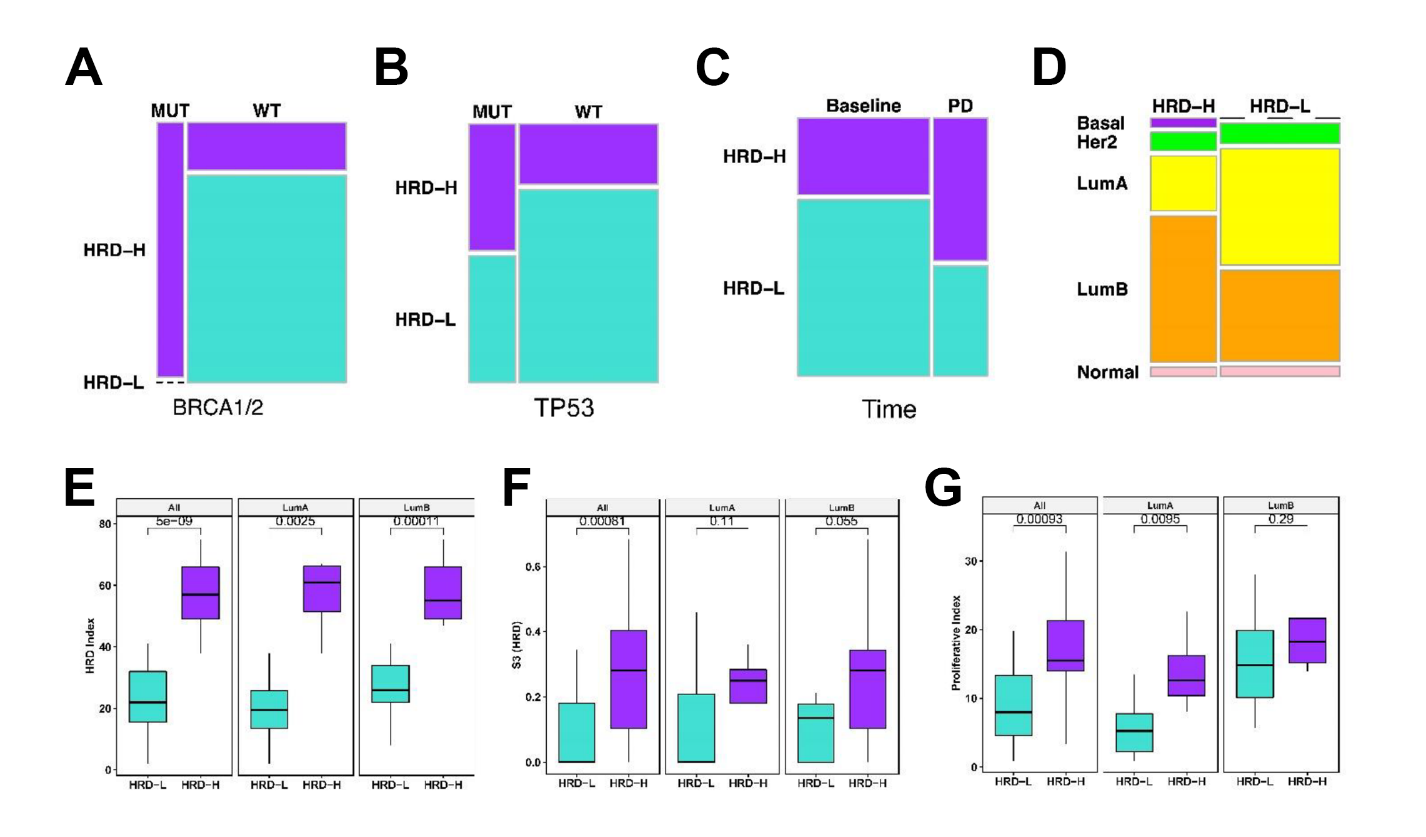


**Figure S3. Characteristics of HRD-high tumors co-occurring with TP53 mutations**

Distributions of HRD index (A), proliferative index (B), estrogen response early (C) and CDKN1A expression (Log2TPM) (D) vs. co-occurrence statuses of TP53 somatic mutation and HRD cluster. HRD: Homologous Recombination Deficiency.


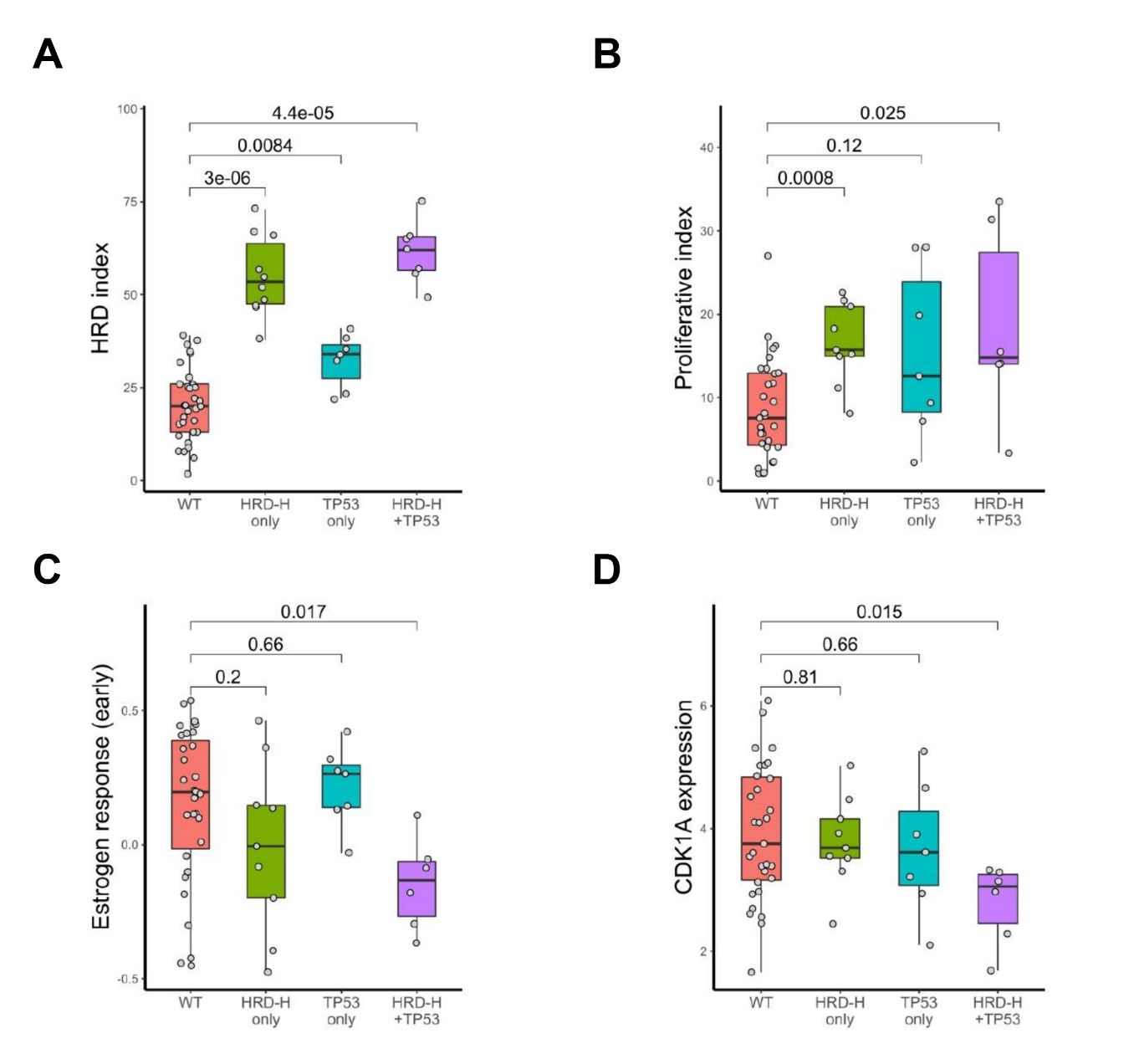


**Figure S4. Kaplan-Meier plots of expression-based prognosis markers**

Kaplan-Meier plots of baseline gene expression for genes that play key roles in cell growth and proliferation - CCNE1 (A), CCNE2 (B) and E1F1 (C). Hazard ratio (HR) with 95% confidence interval (in parenthesis) is shown with corresponding p-values determined by log-rank test. Kaplan-Meier plots of gene expression signatures – HALLMARK E2F targets (D), EGUCHI cell cycle RB1 targets (E) and HALLMARK mTORC1 signaling (F). Kaplan-Meier plots of PGR gene expression (G) and estrogen response early signature in baseline samples (H). Baseline samples were split by median in all plots.


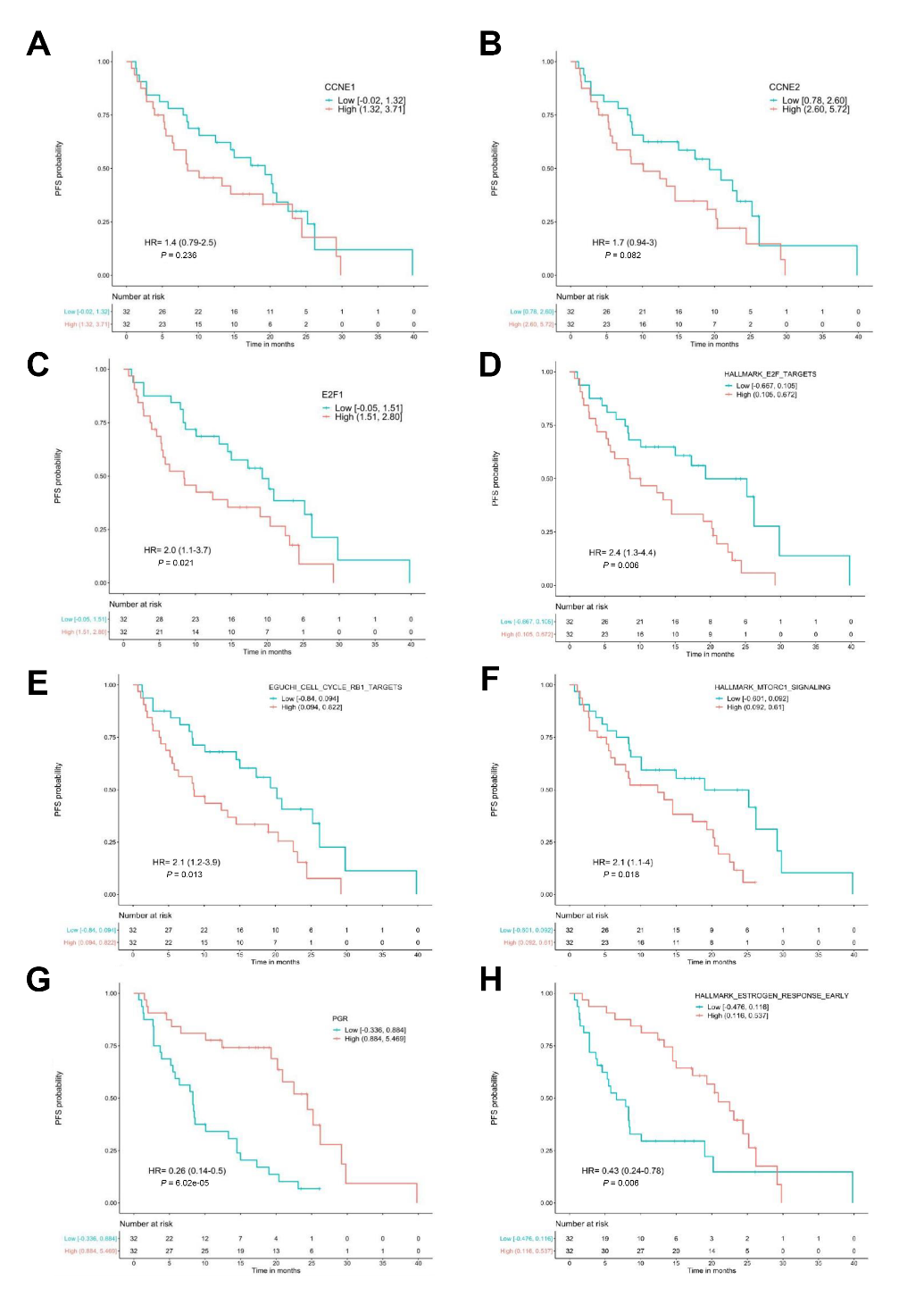


**Figure S5. Proliferative cluster enriched in poor prognostic markers**

(A) Unsupervised clustering of PFS-associated expression signatures. Proliferative cluster: classification of tumor samples harboring lower (C1) or higher (C2) cell growth and proliferation signatures. Time: treatment time. (B) The proliferative cluster C2 has significantly higher proliferative index in the overall cohort and within Luminal A and Luminal B subtypes (Wilcoxon). C2 also has significantly higher HRD index (C) and S3 mutation signature (D). (E) The proliferative cluster C2 is significantly enriched in Luminal B subtype (fisher’s exact test: *p*=1e-5). (F) The proliferative cluster C2 is also enriched in the HRD-H cluster (F), BRCA1/2 pathogenic mutation (G) and TP53 somatic mutation (H) (fisher’s exact test: *p*=0.00091 for HRD cluster; *p*=0.0926 for BRCA1/2 mutation; *p*=0.04428 for TP53 mutation). The proliferative index is significantly associated with worse PFS (I).


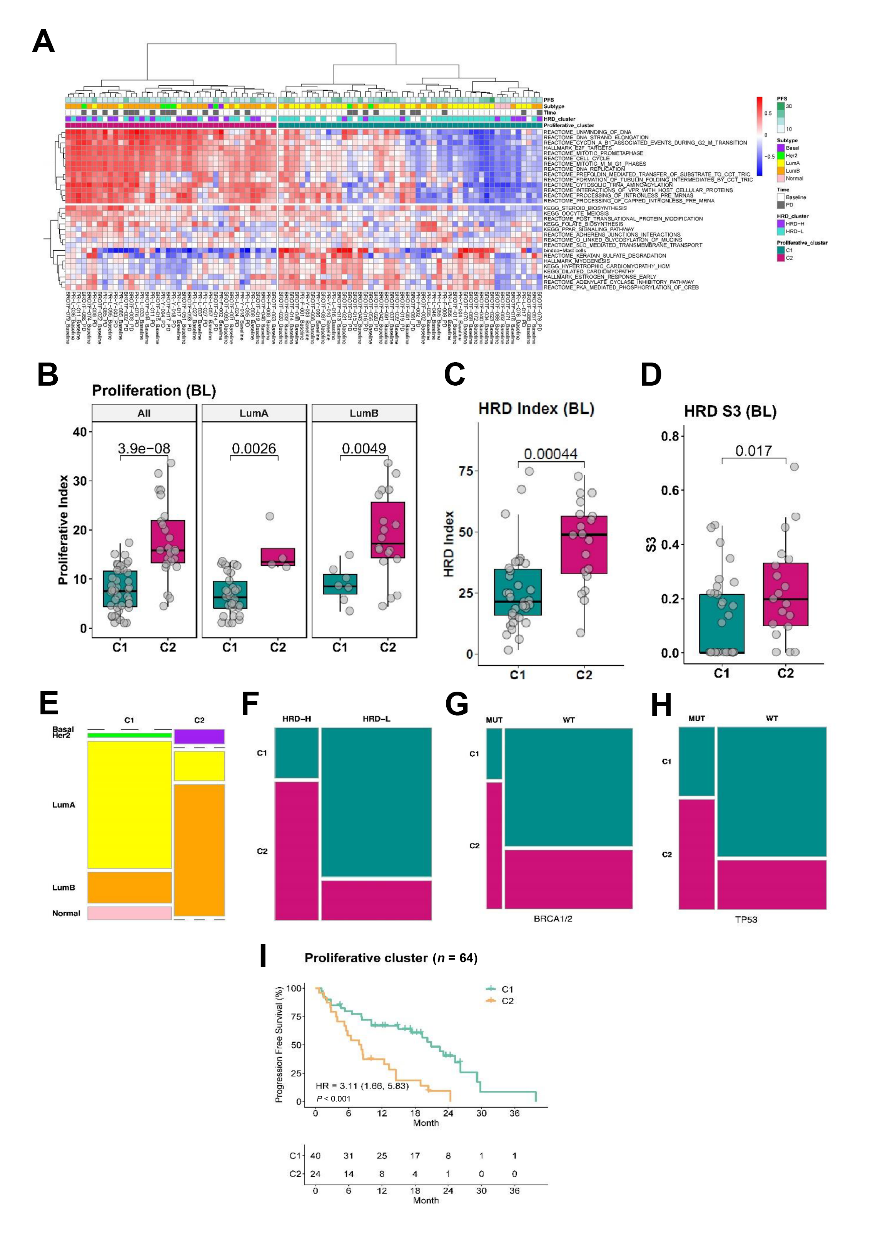


**Figure S6. Integrative analysis identified distinct prognostic clusters**

Clustering pattern of poor prognostic features and key expression signatures (A) based on both baseline and PD samples as grouped by integrative cluster status. (B) Kaplan-Meier plots comparing PFS between four integrative clusters of baseline samples.


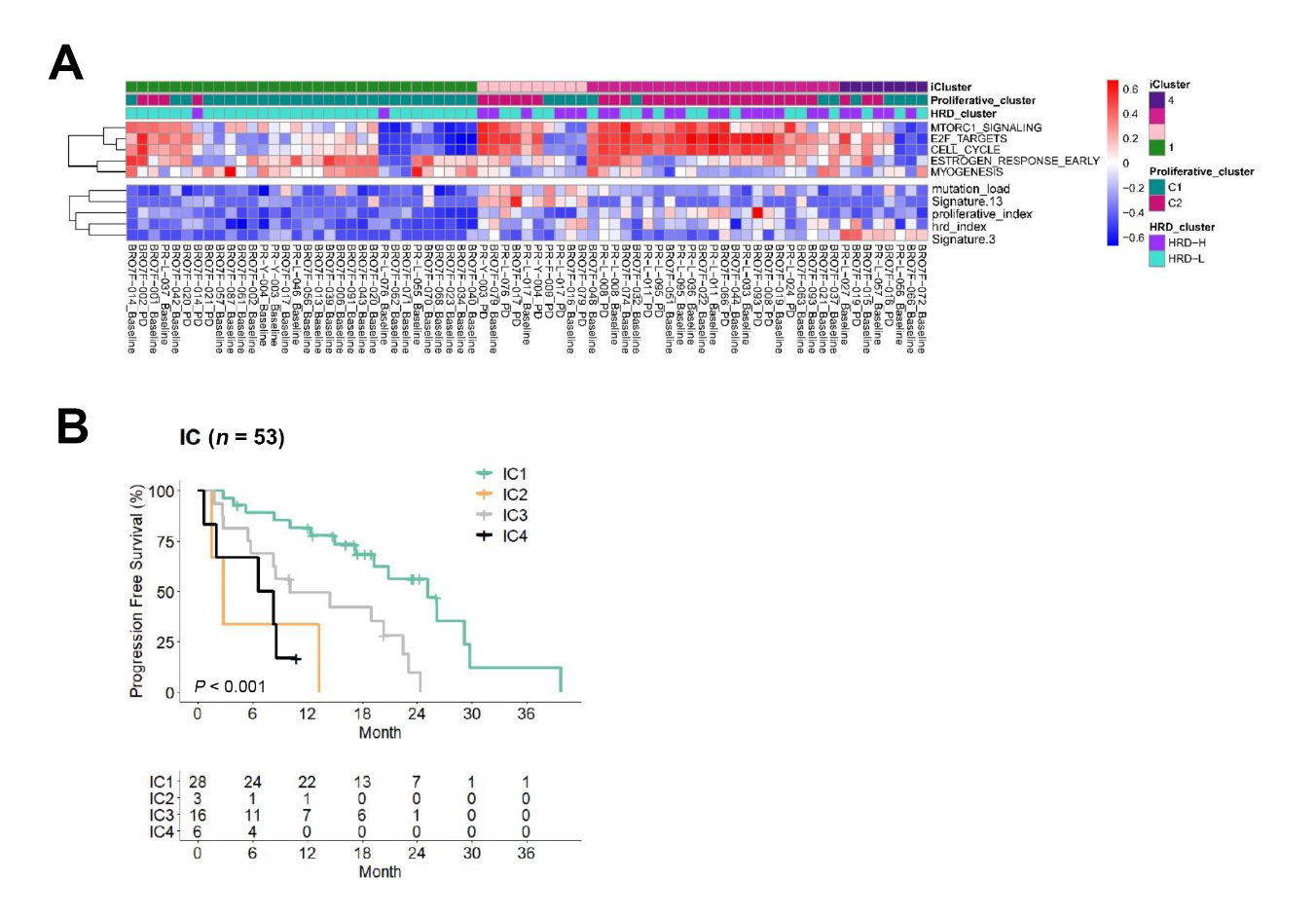


**Figure S7. Molecular characteristics of integrative clusters**

(A) Among baseline samples, the Luminal B subtype is enriched in poor prognostic clusters IC2 & IC3 (fisher’s exact test: *p*=0.00197). The proliferative cluster (B) and the HRD-H cluster (C) are enriched in IC2-4 compared to IC1 (fisher’s exact test: *p*=2e-05 for HRD cluster; *p*=1e-05 for proliferative cluster). (D) Distributions of integrative clusters at BL vs. PD (fisher’s exact test: *p*=0.00506). Distributions of proliferative index (E), estrogen response signature (F), APOBEC S13 (G) and HRD S3 mutation signature (H) vs. integrative cluster status of baseline samples. APOBEC: apolipoprotein B editing complex. BL: baseline. HRD: Homologous Recombination Deficiency. PD: progressive disease


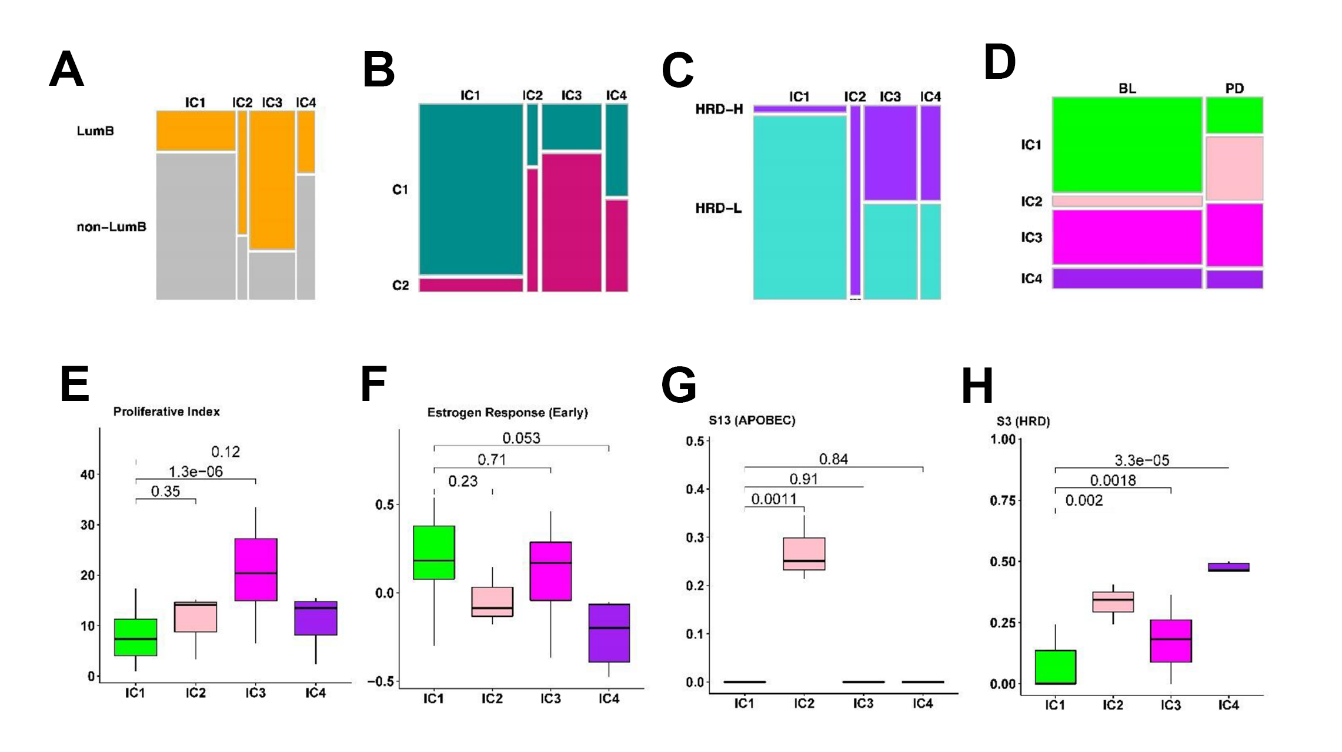


**Figure S8. Subtype switching driven by changes in PAM50 score composition**

PAM50 subtype score compositions of paired BL and PD samples for six cases that switched to the Luminal B subtype (A) and those that switched to the HER2E subtype at PD (B). Pam50.score: PAM50 distance-to-centroid score calculated for each molecular subtype.


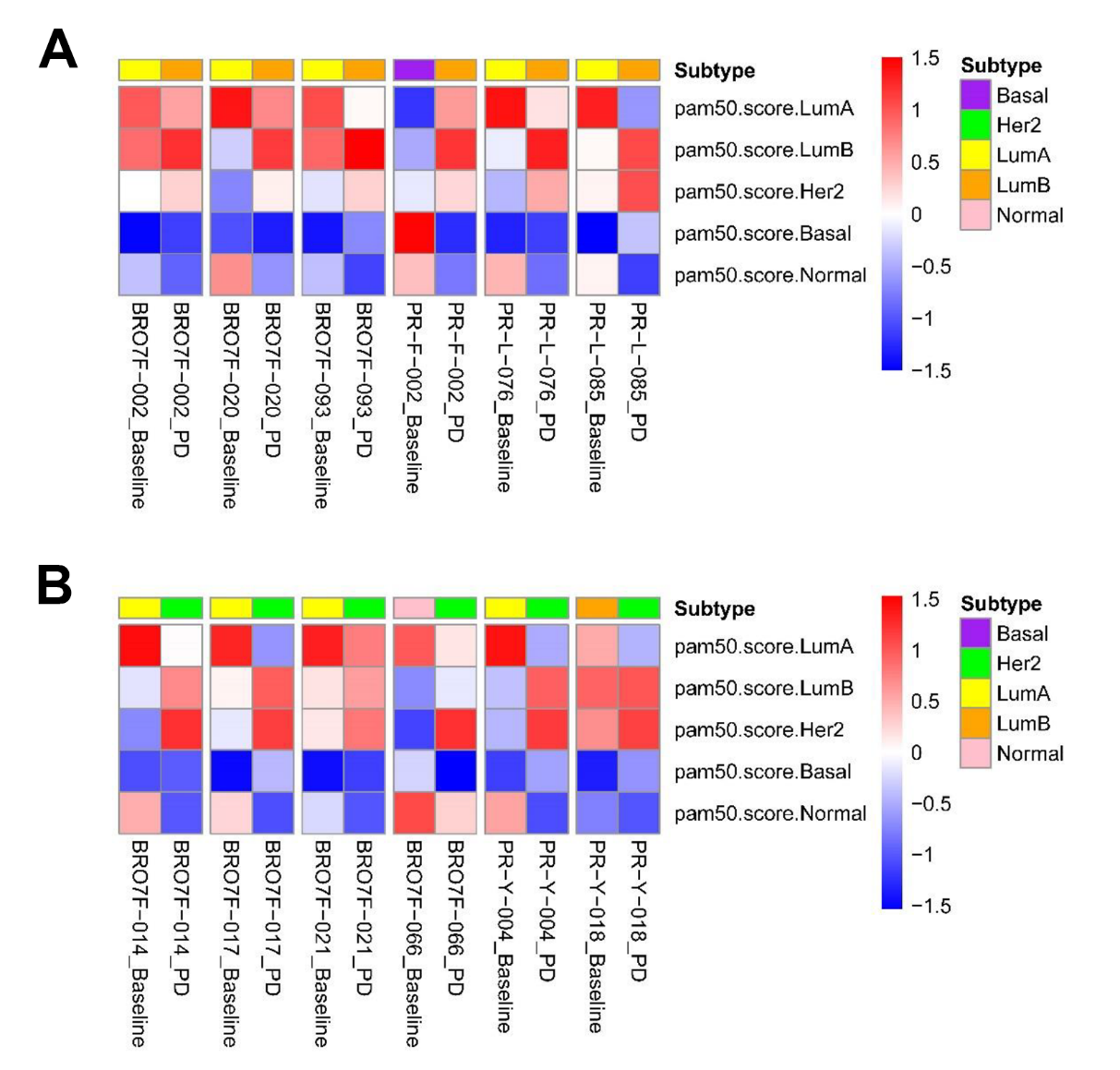


**Figure S9. Increased tumor growth and proliferation at PD**

Comparing the changes in CCNE1, CCNE2 and E2F1 gene expression at BL vs. PD among all samples (A) and longitudinally paired samples (B). Comparing the changes in CCNE1 (C), CCNE2 (D) between paired BL and PD tumors among three groups of patients. All: all patients with paired BL and BD samples. Subtype-Switch: subtypes switched from one subtype at BL to a different subtype at PD. No-Switch: subtypes remained the same between BL and PD. BL: baseline. PD: progressive disease.


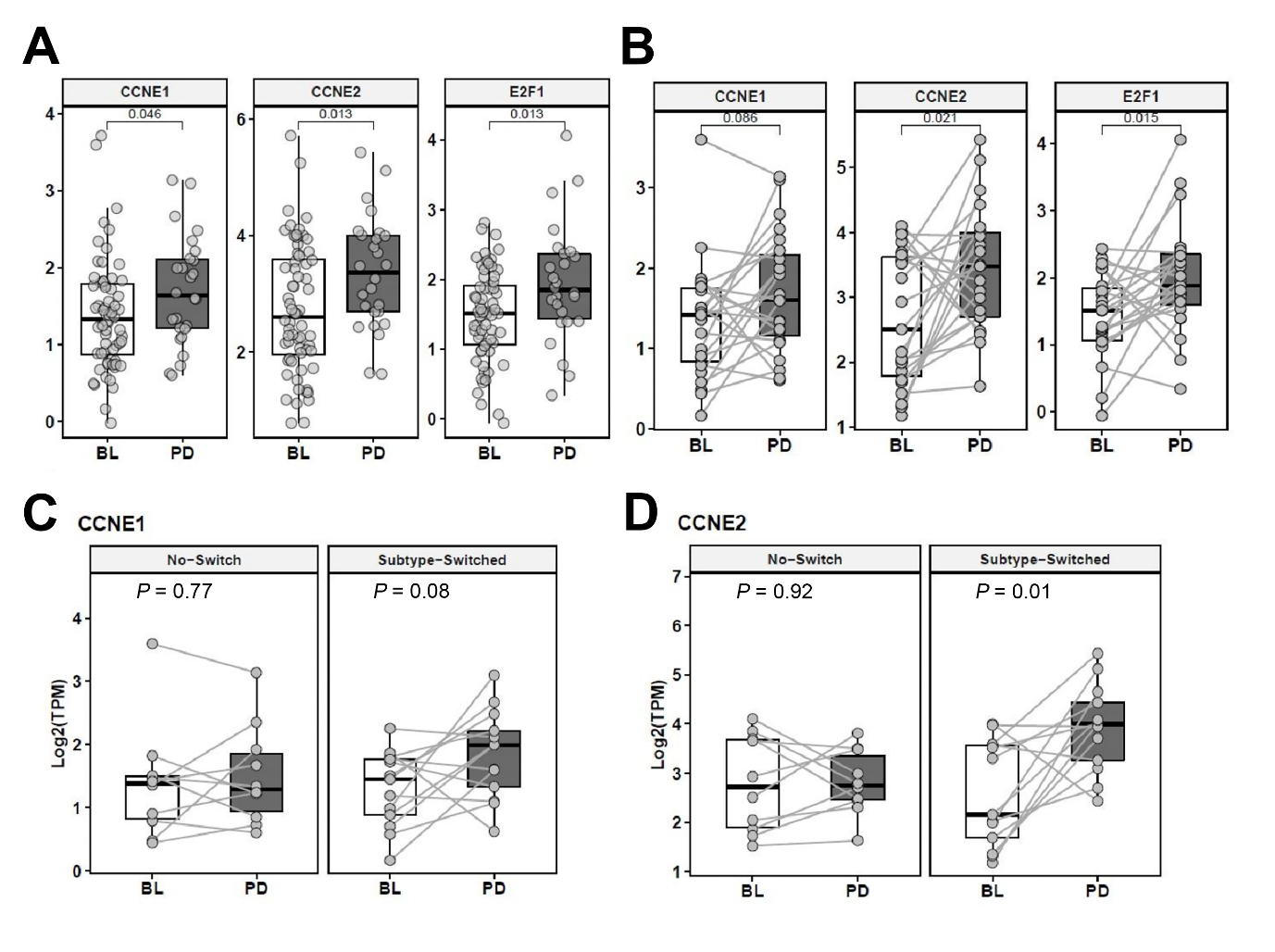


**Figure S10. IHC analysis of cell cycle markers**

Comparing changes in cyclin E1, E2, Ki67 and pRB marker levels between BL and PD for all tumors (A) and longitudinally paired tumors (B) based on IHC analysis. H-score: histopathology metrics of marker positive cell density. (C) Cyclin E1, E2 and Ki67 IHC images of BL and PD tumors from patient BRO7F-014, with BL subtype switched from Luminal A to HER2E at PD.


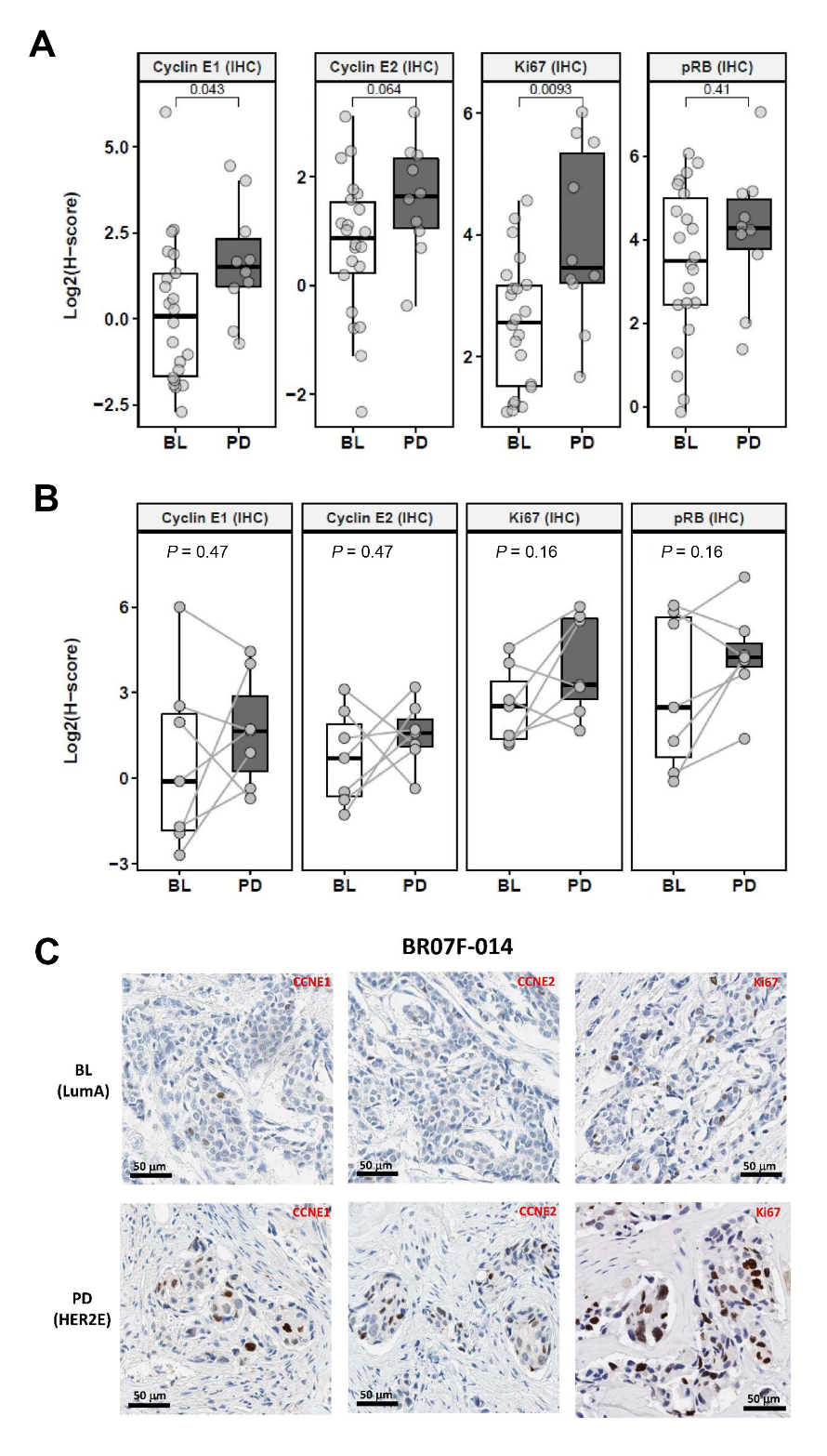


**Figure S11. Landscape of PD-specific genomic alterations**

Mutation diagrams with protein domain annotations for ESR1 genomic alterations (A) and KMT2C somatic mutations (B) detected in the paired BL/PD samples. (A) We observed 12 ESR1 mutations in 8 PD samples and 2 baseline samples. In addition, we identified 2 PD specific ESR fusion events in 2 patients. (B) We observed 3 PD-specific loss of function mutations in 21 patients. In addition, we observed 2 patients with KMT2C mutations in both baseline and PD samples, and 1 patient with mutation in only baseline sample. BL: baseline. PD: progressive disease. (c) We observed 3 PD-specific PTEN mutations in 2 patients and 4 mutations in both paired BL/PD samples from three patients.


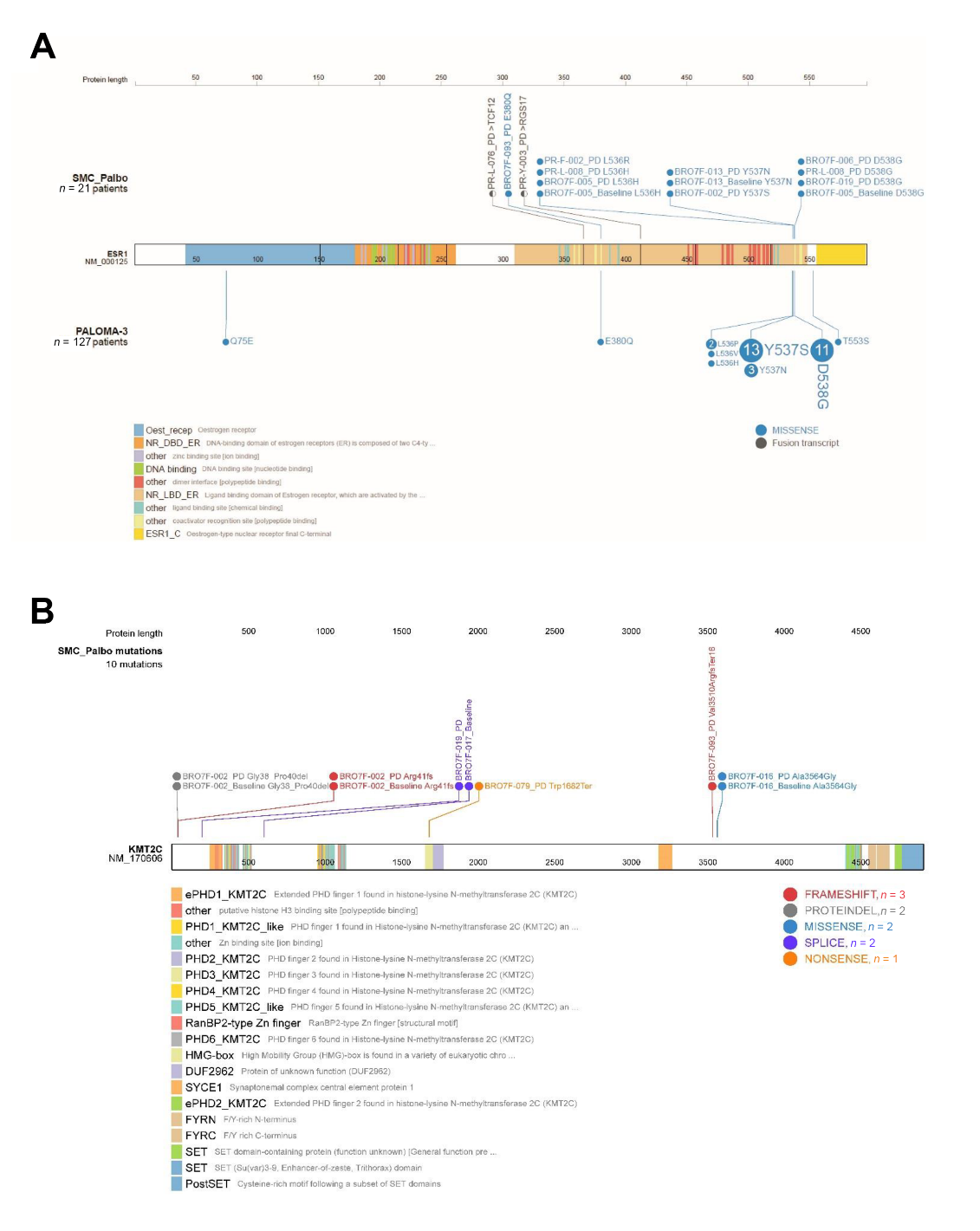


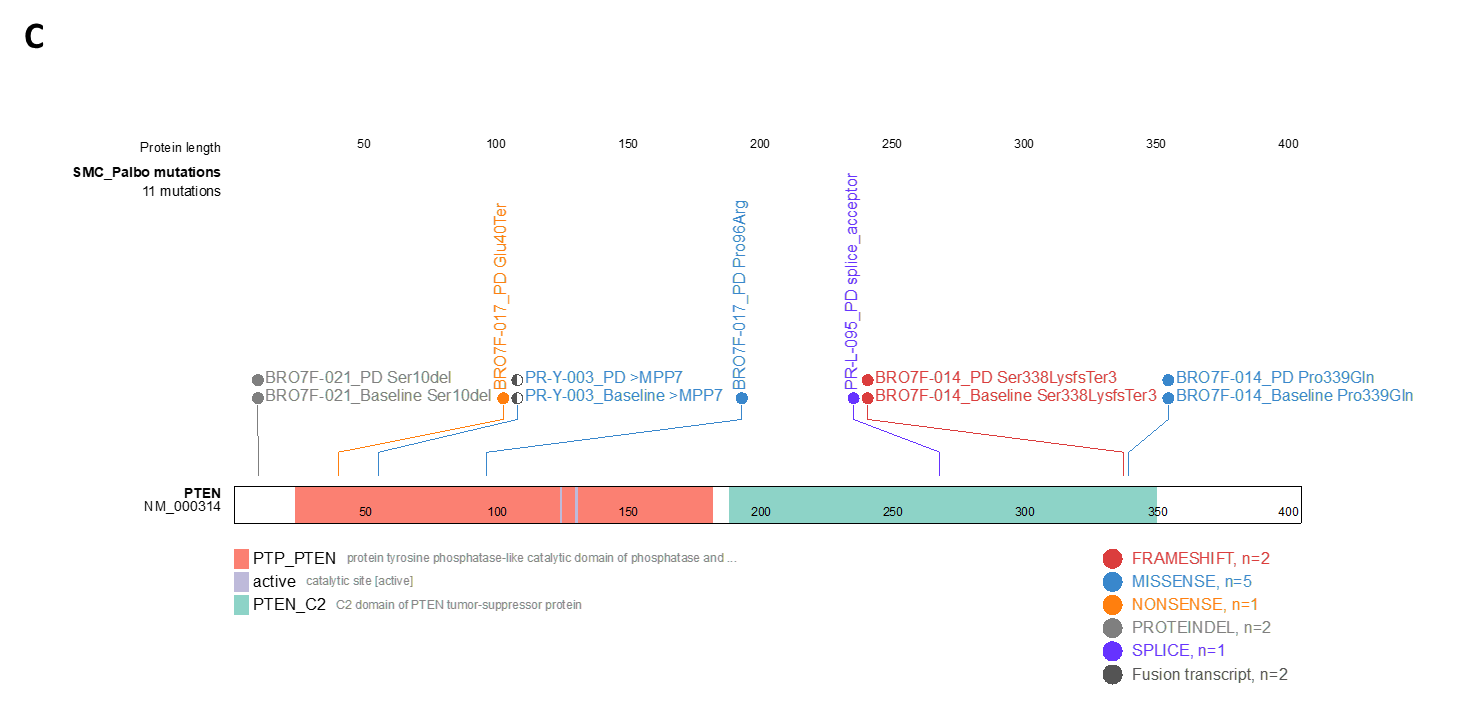


**Figure S12. RB1 LOF associated with increased APOBEC signature at PD**

Comparing longitudinal changes in APOBEC S13 mutation signature (A), REPLICATION STRESS (B), *APOBEC3B* (C) and *CHEK1* (D) gene expression between paired BL and PD tumors among three groups. RB1 LOF: patients harboring PD-specific RB1 loss-of-function genomic alterations at PD. ESR1 GOF: patients harboring PD-specific ESR1 gain-of-function genomic alterations at PD. Other: all other patients with paired BL and PD samples. REPLICATION STRESS: REACTOME ACTIVATION OF ATR IN RESPONSE TO REPLICATION STRESS gene set. APOBEC: apolipoprotein B editing complex. BL: baseline. PD: progressive disease


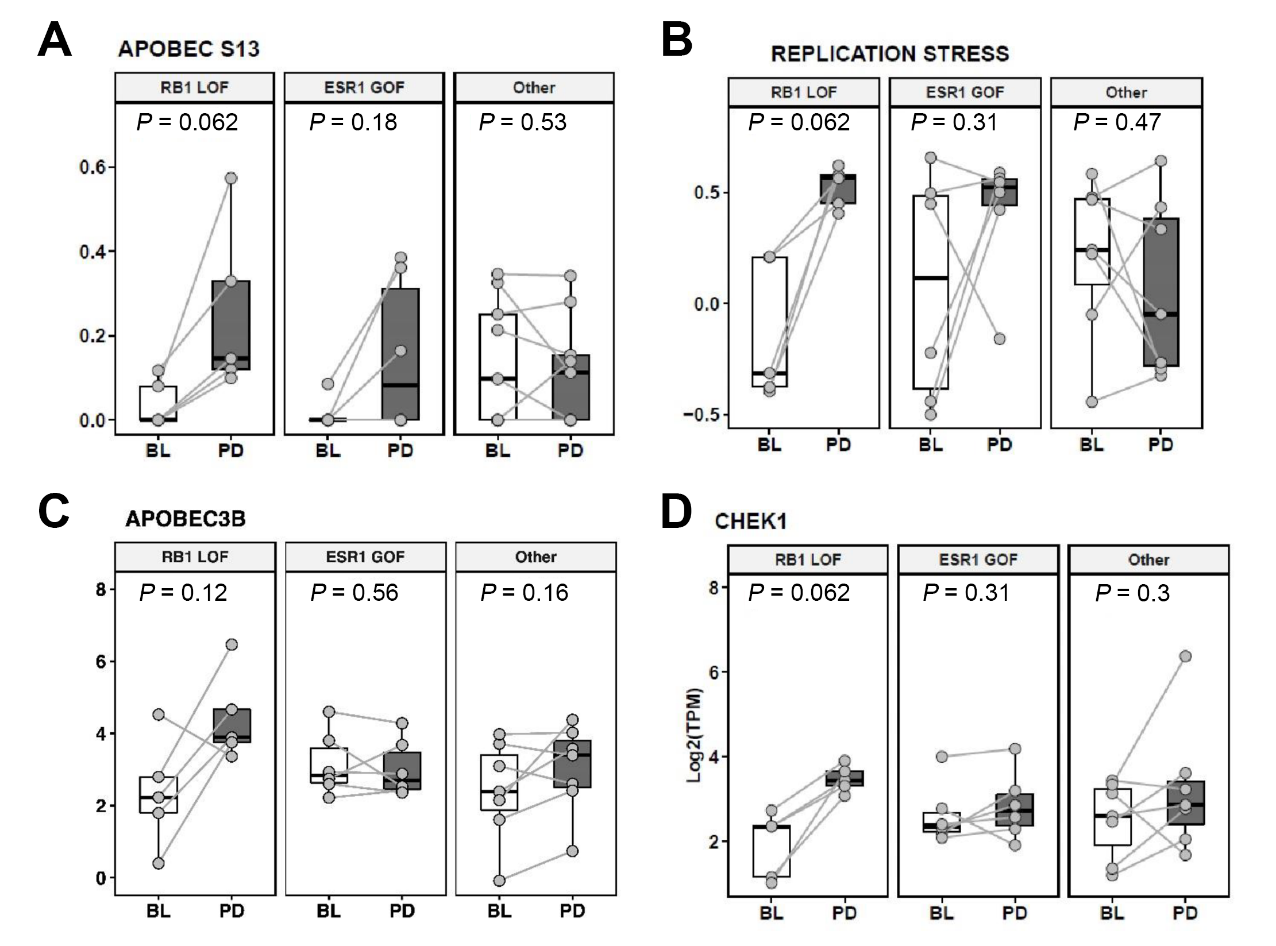


**Figure S13. APOBEC signature enriched in PD-specific tumor subclones**

(A) APOBEC S13 signature contribution in the clone level. All clonal clusters from 21 pairs of baseline and PD were merged and divided into two groups based on PD-specific status (Wilcoxon). Tumor clonal evolution analysis result for paired baseline and PD tumors from patient BRO7F-017: (B) Clonal dynamics over time are shown for baseline and PD tumors. Four tumor subclones were identified from this patient and cancer genes with high-confidence pathogenic mutations are shown in the clones that they originated from. (C) The inferred evolutionary relationships among the tumor subclones are represented by the phylogenic tree, which indicates that several acquired cancer gene mutations including RB1 may drive the emergence and expansion of subclone #3 that is a predominant PD-specific clone. (D) Fractions of mutational signatures identified in the four subclones for patient BRO7F-017. First two stacked bar graphs represent relative contributions of different mutational signatures in the entire baseline tumor and remaining three bar graphs show the contributions among the entire PD tumor. The signatures with less than 10% contribution across all subclones were considered “others.” APOBEC S13 signature was predominant in C3 PD-specific clone. APOBEC: apolipoprotein B editing complex. BL: baseline. PD: progressive disease.
